# Crystal Structures of Xenon(VI) Salts: XeF_5_Ni(AsF_6_)_3_, XeF_5_AF_6_ (A = Nb, Ta, Ru, Rh, Ir, Pt, Au), and XeF_5_A_2_F_11_ (A = Nb, Ta)

**DOI:** 10.3390/molecules28083370

**Published:** 2023-04-11

**Authors:** Zoran Mazej, Evgeny Goreshnik

**Affiliations:** Department of Inorganic Chemistry and Technology, Jožef Stefan Institute, Jamova Cesta 39, SI-1000 Ljubljana, Slovenia; evgeny.goreshnik@ijs.si

**Keywords:** metal, fluorine, xenon, crystal structure, Raman spectroscopy, photochemistry

## Abstract

Experiments on the preparation of the new mixed cations XeF_5_M(AF_6_)_3_ (M = Cu, Ni; A = Cr, Nb, Ta, Ru, Rh, Re, Os, Ir, Pt, Au, As), XeF_5_M(SbF_6_)_3_ (M = Sn, Pb), and XeF_5_M(BF_4_)_x_(SbF_6_)_3-x_ (x = 1, 2, 3; M = Co, Mn, Ni, Zn) salts were successful only in the preparation of XeF_5_Ni(AsF_6_)_3_. In other cases, mixtures of different products, mostly XeF_5_AF_6_ and XeF_5_A_2_F_11_ salts, were obtained. The crystal structures of XeF_5_Ni(AsF_6_)_3_, XeF_5_TaF_6_, XeF_5_RhF_6_, XeF_5_IrF_6_, XeF_5_Nb_2_F_11_, XeF_5_Ta_2_F_11_, and [Ni(XeF_2_)_2_](IrF_6_)_2_ were determined for the first time on single crystals at 150 K by X-ray diffraction. The crystal structures of XeF_5_NbF_6_, XeF_5_PtF_6_, XeF_5_RuF_6_, XeF_5_AuF_6_, and (Xe_2_F_11_)_2_(NiF_6_) were redetermined by the same method at 150 K. The crystal structure of XeF_5_RhF_6_ represents a new structural type in the family of XeF_5_AF_6_ salts, which crystallize in four different structural types. The XeF_5_A_2_F_11_ salts (M = Nb, Ta) are not isotypic and both represent a new structure type. They consist of [XeF_5_]^+^ cations and dimeric [A_2_F_11_]^−^ anions. The crystal structure of [Ni(XeF_2_)_2_](IrF_6_)_2_ is a first example of a coordination compound in which XeF_2_ is coordinated to the Ni^2+^ cation.

## 1. Introduction

The synthesis of XeF_6_ was first described in 1962 [1]. It was prepared by the reaction between xenon and fluorine (molar ratio 1:20) at 700 °C and a pressure of ~200 bar F_2_. Later systematic studies showed that XeF_6_ can be prepared under milder conditions (molar ratio 1:10; 200 °C; and a total pressure of 33 bar) [2]. In general, XeF_6_ is prepared by heating a mixture of Xe and F_2_ (molar ratio 1:20) at 300 °C and a total pressure of ~50 bar [3]. In the presence of a NiF_2_ catalyst, XeF_6_ forms explosively from the gaseous mixture of xenon and fluorine in a molar ratio of 1:5 already at 120 °C [4]. An alternative method with high yield for the preparation of high-purity XeF_6_ is the reaction between Xe and F_2_ at low pressure and high filament temperature in a “hot wire” reactor [5]. At room temperature, XeF_6_ is solid (*T*_m.p._ = 49.48 °C, *T*_b.p._ = 75.57 °C) with a vapor pressure of about 0.03 bar at 23 °C [6]. The color of solid XeF_6_ has been reported to range from colorless to intense yellow. An explanation for these color variations of solid XeF_6_ is not apparent to date [7]. Liquid XeF_6_ and its vapors are yellow-green [6]. There are six, possibly seven, different modifications of solid XeF_6_ [7]. In CF_2_Cl_2_/SO_2_ClF solution, XeF_6_ exists as a tetramer (XeF_6_)_4_ [8], while in the gas phase XeF_6_ exists as a monomer [9,10,11]. The presence of a sterically active free valence electron pair on Xe leads to the XeF_6_ molecule being fluxional [12]. Consequently, the structure of monomeric XeF_6_ has been a major challenge for theoretical computational chemistry [12,13,14,15,16,17]. Experiments have clearly shown that XeF_6_ is not octahedral (*O*_h_) but most likely has the shape of a slightly distorted octahedron (*C*_3v_), naturally in a dynamical form [18]. Both conformers are energetically very close to each other [15]. Because of the low barrier of interconversion, XeF_6_ is a highly fluoxinal molecule that rapidly converts between the 8-fold degenerate *C*_3v_ structures via the octahedral minimum even at low temperatures [15]. In further studies, XeF_6_ was theoretically shown to exhibit a genuine quantum mechanical fluorine tunneling rearrangement, where it “jumps” rapidly between isomers even near 0 K [16]. In solid argon and neon matrices, there are significant interactions between isolated XeF_6_ monomers and the noble gas host [15]. The results of infrared spectroscopy of XeF_6_ in Ne matrix, supported by theoretical calculations, agreed with the *C*_3v_ conformer [15].

Shortly after the first report on the synthesis of XeF_6_, it was found that XeF_6_ is a good fluoride ion donor that reacts with Lewis acids AF_5_ (A = As, Sb, Pt, V, P) to form *n*XeF_6_⋅AF_5_ adducts [19,20,21,22,23]. Crystal structure determination of XeF_6_⋅PtF_5_ revealed a molecular geometry consistent with the ionic formula [XeF_5_]^+^[PtF_6_]^−^ [24]. The compounds XeF_6_⋅2AF_5_ are also [XeF_5_]^+^ salts [25], while the compounds 2XeF_6_⋅AF_5_ are [Xe_2_F_11_]^+^ salts [26,27,28]. The next step was the discovery that XeF_5_AF_6_ salts can bind neutral molecules as XeF_2_ [29,30,31]. Later, this was extended to HF [32], XeOF_4_ [33], and even KrF_2_ [32]. Recently, the XeF_5_SbF_6_ salt was also found to react with other MSbF_6_ and M(SbF_6_)_2_ salts to give XeF_5_M(SbF_6_)_2_ (M = NO_2_^+^, Rb^+^, Cs^+^) [28,34], XeF_5_M(SbF_6_)_3_ (M^2+^ = Mg, Mn, Co, Ni, Cu, Zn, Pd), and (XeF_5_)_3_[M(HF)_2_](SbF_6_)_7_ (M = Hg) [34,35] or even more complex salts (H_3_O)(XeF_5_)_2_M_2_(SbF_6_)_7_∙*n*HF (M = Ca, Cd) [36] and (O_2_)(XeF_5_)_2_Sr_4_(SbF_6_)_11_∙8HF [36] with three different cations. In addition, mixed anion salts Cs[XeF_5_][Bi_x_Sb_1-x_F_6_] [28] and [XeF_5_][As_1-x_Sb_x_F_6_) (x~0.5 and 0.7) [37] have also been reported.

XeF_6_ is a stronger oxidizing and fluorinating agent than XeF_2_ and XeF_4_. Theoretically, XeF_6_ could be used as a fluorinating agent. Unfortunately, XeF_6_ and its XeF_5_^+^ salts are very sensitive to moisture. When they are exposed to water, they hydrolyze and eventually form XeO_3_. The latter is an unstable compound that is extremely sensitive to impact and poses a dangerous explosion hazard when in contact with organic materials. For this reason, XeF_6_ and its XeF_5_^+^ salts currently have no practical significance.

This contribution reports the results of experiments on the preparation of XeF_5_M(SbF_6_)_3_ (M = Sn, Pb), XeF_5_M(BF_4_)_x_(SbF_6_)_3-x_ (x = 1, 2, 3; M = Co, Mn, Ni, Zn), and XeF_5_M(AF_6_)_3_ salts (M = Cu, Ni; A = Cr, Nb, Ta, Ru, Rh, Re, Os, Ir, Pt, Au, As). The experiments were successful only in the preparation of XeF_5_Ni(AsF_6_)_3_, and many other phases were obtained in other experiments. The crystal structures of XeF_5_Nb_2_F_11_, XeF_5_TaF_6_, XeF_5_Ta_2_F_11_, XeF_5_RhF_6_, XeF_5_IrF_6_, and Ni(XeF_2_)_2_(IrF_6_)_2_ were determined for the first time. The crystal structures of XeF_5_NbF_6_ [30], XeF_5_PtF_6_ [24], XeF_5_RuF_6_ [38], XeF_5_AuF_6_ [39], and (Xe_2_F_11_)_2_(NiF_6_) [40] were redetermined with higher accuracy than previously reported.

## 2. Results

### 2.1. Attempted Preparation of the Salts XeF_5_M(AF_6_)_3_ (M = Cu, Ni; A = Cr, Nb, Ta, Ru, Rh, Re, Os, Ir, Pt, Au, As), XeF_5_M(SbF_6_)_3_ (M = Sn, Pb), and XeF_5_M(BF_4_)_x_(SbF_6_)_3-x_ (x = 1, 2, 3; M = Co, Mn, Ni, Zn)

The proposed synthetic methods lead to a mixture of substances. The work carried out is an X-ray diffraction study of some crystal phases of these mixtures. In addition, some of the products were also confirmed by Raman spectroscopy (Supplementary Materials).

Reactions between XeF_2_, MF_2_ (M = Cu, Ni), AsF_5_, and UV-irradiated F_2_ in anhydrous hydrogen fluoride (aHF) resulted in clear colorless (Cu) and yellow (Ni) solutions (Table S1). In the case of nickel, single crystals of XeF_5_Ni(AsF_6_)_3_ were obtained upon crystallization (Table 1), while in the case of copper a mixture of single crystals of XeF_5_AsF_6_ [41] and CuFAsF_6_ [42] was observed in the crystallization product. In all other experiments where mixtures of XeF_2_/MF_2_ (M = Ni, Cu) with addition of AF_3_ (A = Cr, Au), AF_5_ (A = Nb, Ta), or metal powder A (A = Re, Ru, Rh, Os, Ir, Pt) were treated with UV-irradiated F_2_ (Table S1), the insoluble material did not disappear even after several days. For crystallization, the clear supernatant, which contained no visible sediments, was decanted into the side arm of the double-arm crystallization vessel. Only single crystals of XeF_5_AF_6_ (A = Nb, Ta, Ru, Rh, Ir, Pt, Au) and XeF_5_A_2_F_11_ salts (A = Nb, Ta) were grown from the corresponding solutions (Table S1). In the case of Ru and Pt, traces of O_2_AF_6_ salts (A = Ru, Pt) [43,44] were also present. Although the remaining insoluble solids were not characterized, it can be assumed that they probably consisted of M(AF_6_)_2_ salts (M = Ni, Cu; A = Nb, Ta, Ru, Rh, Ir, Pt, Au). Of these, only Cu(AuF_6_)_2_ and Ni(AuF_6_)_2_ [45] are known, while the others have not yet been synthesized. Similar to the M[AuF_6_]_2_ salts, unlike the M(AF_6_)_2_ salts (A = As, Sb) [46], they are probably not well soluble or they are insoluble in anhydrous HF. For the M(AF_6_)_2_ salts (M = Ni, Cu; A = Nb, Ta, Ru, Rh, Os, Ir, Pt), their lattice energy appears to overcome the solvation energy. This would explain their insolubility in aHF and the preferential formation of mixtures of M(AF_6_)_2_ (insoluble in aHF) and XeF_5_AF_6_ (soluble in aHF) instead of XeF_5_M(AF_6_)_3_ salts (M = Cu, Ni; A = Nb, Ta, Ru, Rh, Ir, Pt, Au).

In the XeF_2_/NiF_2_/Ir/UV-irradiated F_2_/aHF system single crystals of XeF_5_IrF_6_ and Ni(XeF_2_)_2_(IrF_6_)_2_ were found in the same batch after crystallization (Table S1). This means that either the F_2_ concentration was too low to oxidize all of the Xe(II) to Xe(VI), or that partial reduction of Xe(VI) occurred during crystallization.

In the XeF_2_/NiF_2_/Re/UV-irradiated F_2_ system, few single crystals of (Xe_2_F_11_)_2_(NiF_6_) were detected as crystallization product (Table S1). When pure Re powder is treated with UV-irradiated F_2_ in aHF, it oxidizes to volatile ReF_6_ [47]. The latter does not react in aHF even with a very good fluorine ion donor such as CsF [47]. In the XeF_2_/NiF_2_/Re/UV-irradiated F_2_/aHF system, all the Re is oxidized to inert gaseous ReF_6_. When the NiF_2_/F_2_ reaction mixture in aHF is irradiated with UV light, the particles of pale yellow-green NiF_2_, which is insoluble in aHF, turn black. This indicates that NiF_2_ is first fluorinated to NiF_2+x_ (x ≤ 1) [47]. When only XeF_2_ (Xe^II^) is present without other compounds, XeF_2_ is oxidized to Xe^IV^ (XeF_4_) by elemental fluorine under UV light in aHF [47]. Our experiment has shown that in the presence of Ni(II) and Xe(II) as reagents, oxidation to Ni(IV) and Xe(VI) occurs, giving (Xe_2_F_11_)_2_(NiF_6_) [40]. The A’_2_NiF_6_ salts (A’ = Li, Na, K, Cs) can also be prepared by oxidation of NiF_2_ at about 20 °C by sunlight or UV-irradiated F_2_ in liquid aHF containing dissolved alkali metal fluorides (LiF, NaF, KF, CsF) [48].

In the XeF_2_/CuF_2_/Os/UV-irradiated F_2_/HF system, a colorless solution was observed over white insoluble material. Upon cooling the reaction vessel to 77 K, a strong yellow coloration of the solid aHF was observed, indicating the presence of yellow OsF_6_ [49]. When crystallized from the decanted clear solution, only two small colorless crystals were formed. One of them was detected as XeF_4_ by Raman spectroscopy, while the other exploded on the diffractometer goniometer.

An attempt to prepare XeF_5_Cu(CrF_6_)_3_ or to detect the formation of XeF_5_CrF_6_ salt was unsuccessful. Crystallization yielded only single crystals of (XeF_5_CrF_5_)_4_⋅XeF_4_ [50]. Although A’CrF_6_ salts (A’ = Na, K, Rb, Cs) are known [51], the salt XeF_5_^+^CrF_6_^−^ is not. The reaction between CrF_5_ and XeF_6_ proceeds at room temperature in aHF with release of fluorine and formation of XeF_5_CrF_5_ [52]. In the presence of XeF_4_, the very stable (XeF_5_CrF_5_)_4_⋅XeF_4_ is formed [50].

Attempts to prepare XeF_5_M(SbF_6_)_3_ salts (M = Sn, Pb) failed (Table S1). The crystals grown from clear decanted solutions corresponded mainly to XeF_5_Sb_2_F_11_ [25] and XeF_5_SbF_6_ [25]. Various approaches to prepare XeF_5_M(BF_4_)_x_(SbF_6_)_3-x_ salts (x = 1, 2, 3; M = Co, Mn, Ni, Zn) also failed. In the case of Co and Mn, oxidation of M(II) to M(III) occurred, while only crystals of XeF_5_SbF_6_ and XeF_5_Sb_2_F_11_ were found between powdered material. In an attempt to prepare (XeF_5_)Ni(BF_4_)_3_, only single crystals of XeF_5_BF_4_ [28] were found (Table S1).

### 2.2. Crystal Structures of the Salts XeF_5_AF_6_ (A = Nb, Ta, Ru, Rh, Ir, Pt, Au), XeF_5_A_2_F_11_ (A = Nb, Ta), (Xe_2_F_11_)_2_(NiF_6_)_2_, and Ni(XeF_2_)_2_(IrF_6_)_2_

Crystal structures were determined on single crystals by X-ray diffraction. Details of the data acquisition parameters and other crystallographic information for the salts XeF_5_AF_6_ (A = Nb, Ta, Ru, Rh, Ir, Pt, Au), XeF_5_A_2_F_11_ (A = Nb, Ta), and (Xe_2_F_11_)_2_(NiF_6_)_2_ and Ni(XeF_2_)_2_(IrF_6_)_2_ are given in Table 1 and Table 2. The crystal structures of XeF_5_NbF_6_ [30], XeF_5_PtF_6_ [24], XeF_5_RuF_6_ [38], XeF_5_AuF_6_ [39], and (Xe_2_F_11_)_2_(NiF_6_) [40] were redetermined at low temperature with higher accuracy than previously reported.

The crystal structures of XeF_5_AF_6_ (A = Nb, Ta, Ru, Ir, Pt, Sb) are isotypic. The crystal structures of XeF_5_AsF_6_ and XeF_5_AuF_6_ are also isotypic, but differ from the previous structures. The crystal structure of XeF_5_RhF_6_ is a unique representative of a new type of structure. Some geometric parameters of the XeF_5_AF_6_ salts are listed in Table 3 and Table 4.

The crystal structures of XeF_5_Nb_2_F_11_ and XeF_5_Ta_2_F_11_ are not isotypic and they also differ from the previously known crystal structure of XeF_5_Sb_2_F_11_ [25]. Some geometric parameters are listed in Table 5.

The crystal structure of XeF_5_Ni(AsF_6_)_3_ is isotypical to the previously reported crystal structures of XeF_5_M(SbF_6_)_3_ (M^2+^ = Mg, Mn, Co, Ni, Cu, Zn, Pd) [34,35]. Some geometric parameters are given in Table 6.

The crystal structure of [Ni(XeF_2_)_2_](IrF_6_)_2_ is isotypical of crystal structure of [Cu(XeF_2_)_2_](SbF_6_)_2_ reported previously [54]. Some geometric parameters are listed in Table 7.

## 3. Discussion

### 3.1. Crystal Structures of XeF_5_AF_6_ (A = Nb, Ta, Ru, Rh, Os, Ir, Pt, Au, As, Sb)

Prior to this study, the crystal structures of XeF_5_NbF_5_ (293 K) [30], XeF_5_RuF_5_ (RT; room temperature) [38], XeF_5_PtF_5_ (RT) [24], XeF_5_AuF_5_ (RT) [39], XeF_5_AsF_6_ (80 K, 100 K, 150 K, 298 K) [37,41,53], XeF_5_SbF_6_ (150 K, 296 K) [25,37], and mixed anion [XeF_5_][As_0.3_Sb_0.7_F_6_] (200 K, 295 K) and [XeF_5_][As_0.5_Sb_0.5_F_6_] (150 K, 295 K) salts [37] were known. X-ray powder diffraction (XPD) images showed that XeF_5_AF_6_ (A = Os, Ir, Pt, and Ru) are isotypic [39].

The crystal structures of XeF_5_AF_5_ (A = Nb, Ru, Pt, Au) determined at 150 K are the same as at room temperature. The crystal structure of XeF_5_IrF_5_ agrees with the XPD data [39], while the crystal structures of XeF_5_TaF_6_ and XeF_5_RhF_6_ have been determined for the first time. Based on the results of this study and the data known from the literature, the crystal structures of the XeF_5_AF_6_ salts (A = Nb, Ta, Ru, Rh, Os, Ir, Pt, Au, As, Sb) can be classified into four types of structures (type I, II, III, and IV).

#### 3.1.1. Type I; XeF_5_AF_6_ (A = Nb, Ta, Ru, Os, Ir, Pt, Sb) Salts

**Figure 1:**
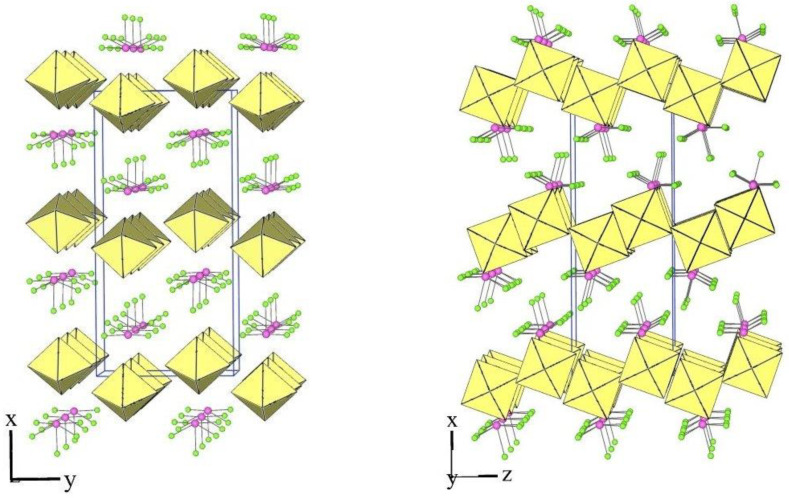
Two different views of the packing of [XeF_5_]^+^ cations and [IrF_6_]^−^ anions in the crystal structure of [XeF_5_][IrF_6_] (type I).

The crystal structure of XeF_5_PtF_6_ was described as the first example of type I [24]. The crystal structures of the salt XeF_5_AF_6_ (A = Nb, Ta, Ru, Os, Ir, Sb) are isotypic to this type (Table 1 and Table 3). The members of type I crystallize in the orthorhombic Pnma space group, in which the asymmetric structural unit consists of a crystallographically unique [XeF_5_]^+^ cation and [AF_6_]^−^ anion (Figure 1).

**Figure 2:**
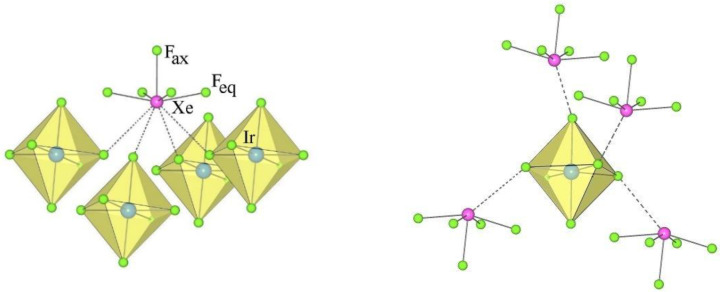
Secondary contacts between the [XeF_5_]^+^ cations and [IrF_6_]^−^ anions in the crystal structure of [XeF_5_][IrF_6_] (type I).

Each [XeF_5_]^+^ cation exhibits the typical geometry, i.e., a pseudo-octahedral AX_5_E VSEPR arrangement of the bond pairs (X) and the lone pair (E). The Xe–F_ax_ bonds are shorter than the other four Xe–F_eq_ distances (Table 4, Figure 2). Each XeF_5_ unit forms four secondary contacts with the fluorine atoms of four AF_6_ groups (Figure 2). Each [AF_6_]^−^ anion participates in four secondary contacts with four different XeF_5_ groups (Figure 2). 

#### 3.1.2. Type II; XeF_5_AF_5_ (A = As, Au) Salts

**Figure 3:**
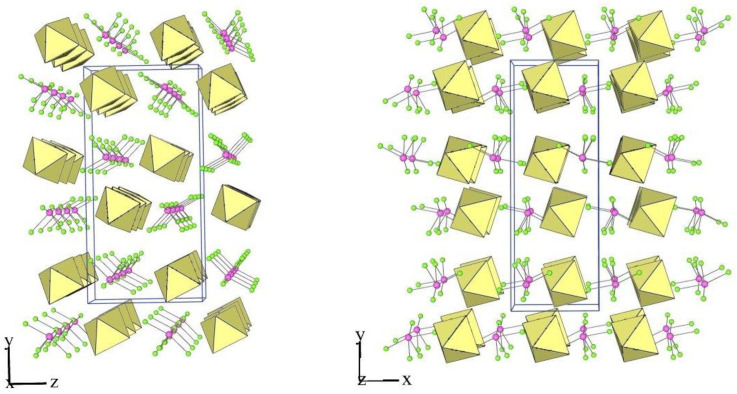
Two different views of the packing of [XeF_5_]^+^ cations and [AuF_6_]^−^ anions in the crystal structure of [XeF_5_][AuF_6_] (type II).

The crystal structures of XeF_5_AuF_6_ and the monoclinic form of XeF_5_AsF_6_ are examples of type II (Table 1 and Table 4). They crystallize in the monoclinic space group *P*2_1_*/c*. The asymmetric structural unit of type II consists of a crystallographically equivalent [XeF_5_]^+^ cation and an [AF_6_]^−^ anion (Figure 3).

**Figure 4:**
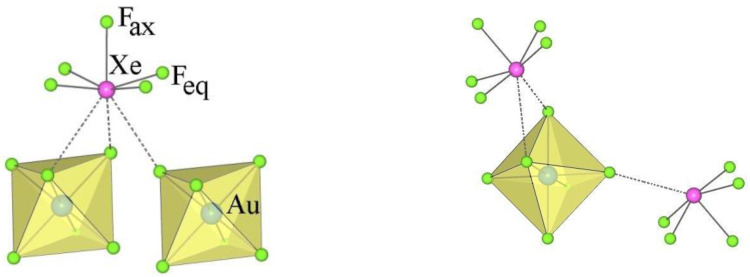
Secondary contacts between the [XeF_5_]^+^ cations and [AuF_6_]^−^ anions in the crystal structure of [XeF_5_][AuF_6_] (type II).

The geometry of the [XeF_5_]^+^ in the salts of type II is the same as in type I [*d*(Xe–F_ax_) < *d*(Xe–F_eq_); Table 4, Figure 4), while the number of secondary contacts between [XeF_5_]^+^ cations and [AF_6_]^−^ anions is different. In type II, each [XeF_5_]^+^ cation forms three secondary contacts with the fluorine atoms of two AF_6_ groups and each [AF_6_]^−^ anion participates in three secondary contacts with two different XeF_5_ groups (Figure 4). 

#### 3.1.3. Type III; XeF_5_AF_5_ (A = Rh) Salts

**Figure 5:**
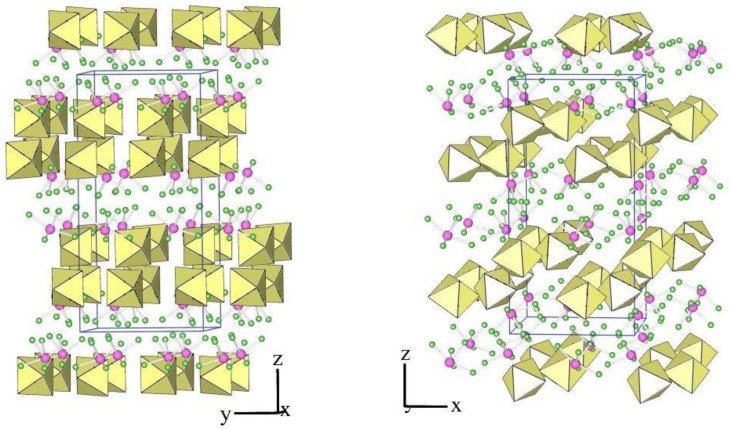
Two different views of the packing of [XeF_5_]^+^ cations and [RhF_6_]^−^ anions in the crystal structure of [XeF_5_][RhF_6_] (type III).

The crystal structure of XeF_5_RhF_6_ is the only representative of type III (Table 1 and Table 4). It crystallizes in the orthorhombic space group *Pbca*. The asymmetric structural unit of type III consists of a crystallographically equivalent [XeF_5_]^+^ cation and an [AF_6_]^−^ anion (Figure 5).

**Figure 6:**
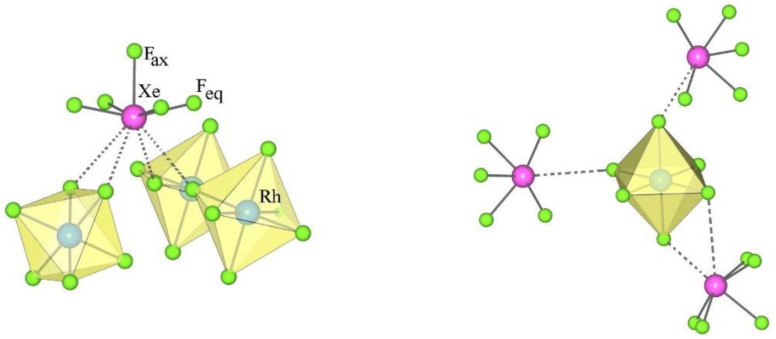
Secondary contacts between the [XeF_5_]^+^ cations and [RhF_6_]^−^ anions in the crystal structure of [XeF_5_][RhF_6_] (type III).

The geometry of the [XeF_5_]^+^ in the type III salts is similar to that in the type I and II compounds (*d*(Xe–F_ax_) < *d*(Xe–F_eq_); Table 4, Figure 6), while the nature of the secondary interaction contacts between the [XeF_5_]^+^ cations and the [AF_6_]^−^ anions is different. In type III, each [XeF_5_]^+^ cation forms four secondary contacts with the fluorine atoms of three AF_6_ groups, while each [AF_6_]^−^ anion participates in four secondary contacts with three different XeF_5_ groups (Figure 6).

#### 3.1.4. Type IV; Orthorhombic XeF_5_AsF_6_ and Mixed Anionic [XeF_5_][As_0.3_Sb_0.7_F_6_) and [XeF_5_][As_0.5_Sb_0.5_F_6_) Salts

The crystal structure of XeF_5_AsF_6_ was first determined at room temperature [41] and later redetermined at 150 K [37]. When the crystal first measured at 150 K was cooled to 80 K and data were collected, no phase transition was observed [37]. In both cases, only a monoclinic phase was obtained (150 K; *P*2_1_/*c*, *Z* = 4, *a* = 5.8222 (6) Å, *b* = 16.3566 (15) Å, *c* = 7.9247 (8) Å, *β* = 90.729 (9) ^o^). However, single crystals of orthorhombic XeF_5_AsF_6_ (Table 8) crystallized from aHF solution between 22 and −30 °C [53]. The corresponding aHF solution was prepared by redox decomposition of [FKrFXeF][AsF_6_]⋅0.5KrF_2_⋅2HF heated stepwise and four different times, from −65 °C to 22 °C. The asymmetric structural unit consists of two crystallographically nonequivalent [XeF_5_]^+^ cations and two [AsF_6_]^−^ anions [53].

Similar unit cell parameters (Table 8) were determined for orthorhombic [XeF_5_][As_0.3_Sb_0.7_F_6_] (100–295 K) and *β*-[XeF_5_][As_0.5_Sb_0.5_F_6_] (295 K), both of which have a (3 + 1)-dimensional incommensurately modulated crystal structure (superspace group *Ama2(00γ)s0s*) [37]. At 150 K, the *α*-[XeF_5_][As_0.5_Sb_0.5_F_6_] salt is also orthorhombic, but not modulated (space group *Pca2_1_*) and with doubled *c*-axis (Table 8) [37]. In [XeF_5_][As_0.3_Sb_0.7_F_6_] and *β*-[XeF_5_][As_0.5_Sb_0.5_F_6_] there are two crystallographically nonequivalent [XeF_5_]^+^ cations and two crystallographically independent sites for pnictogen atoms, while in *α*-[XeF_5_][As_0.5_Sb_0.5_F_6_] there are four crystallographically nonequivalent [XeF_5_]^+^ cations and four crystallographically different sites for pnictogen atoms.

**Figure 7:**
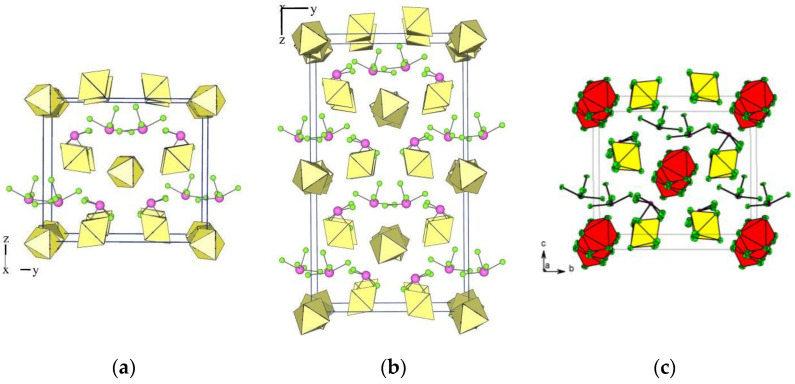
Packing of [XeF_5_]^+^ cations and [AF_6_]^−^ anions (A = As, Sb) in the crystal structures of (**a**) orthorhombic [XeF_5_][AsF_6_]; (**b**) *α*-[XeF_5_][As_0.5_Sb_0.5_F_6_]; (**c**) average structure of [XeF_5_][As_0.3_Sb_0.7_F_6_]. The last figure is reproduced from Ref. [37] and published under the terms and conditions of the Creative Commons Attribution 4.0 International License CC BY 4.0.

All the compounds listed in Table 8 are structurally related, as indicated by the similar packing in their crystal structures (Figure 7). It is practically identical in the orthorhombic XeF_5_AsF_6_, [XeF_5_][As_0.3_Sb_0.7_F_6_], and *β*-[XeF_5_][As_0.5_Sb_0.5_F_6_] and slightly different in *α*-[XeF_5_][As_0.5_Sb_0.5_F_6_] due to the different inclination of some AF_6_ (A = As, Sb)octahedra.

**Figure 8:**
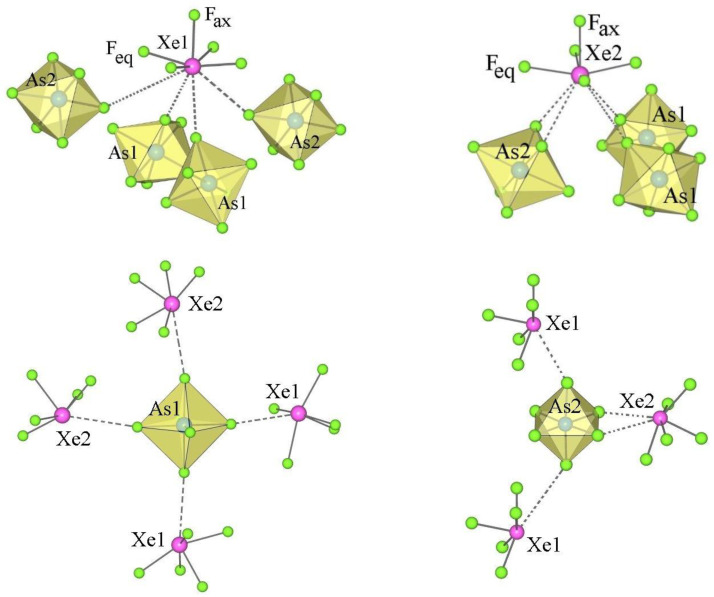
Secondary contacts between the [XeF_5_]^+^ cations and the [AsF_6_]^−^ anions in the crystal structure of orthorhombic [XeF_5_][AsF_6_] (type IV).

In the orthorhombic [XeF_5_][AsF_6_] [53] each [Xe(1)F_5_]^+^ cation forms three shorter (<3 Å) and one longer secondary contact (3.451 (7) Å) with the fluorine atoms of four AsF_6_ groups. The sum of the Xe···F van der Waals radii is 3.63 Å [55]. The other [Xe(2)F_5_]^+^ cation is also involved in four secondary interactions (<3 Å), but only with three AsF_6_ groups. Each [As(1)F_6_]^−^ anion interacts with four [XeF_5_]^+^ cations and each [As(2)F_6_]^−^ anion interacts with only three [XeF_5_]^+^ cations (Figure 8).

**Figure 9:**
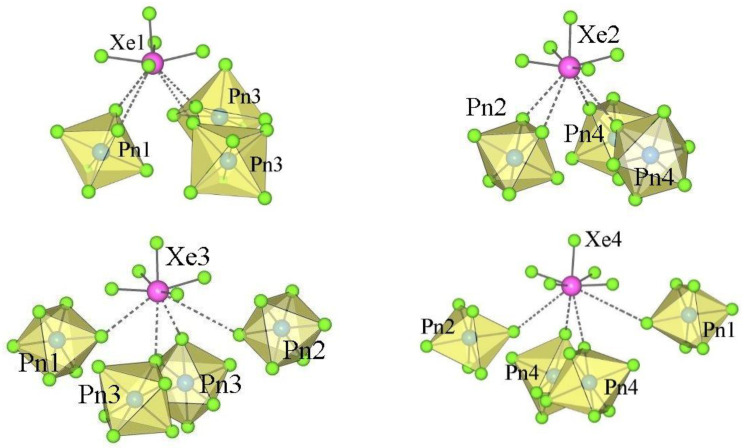
Secondary contacts between the [XeF_5_]^+^ cations and the surrounding [AF_6_]^−^ anions (A = As, Sb) in the crystal structure of *α*-[XeF_5_][As_0.5_Sb_0.5_F_6_].

**Figure 10:**
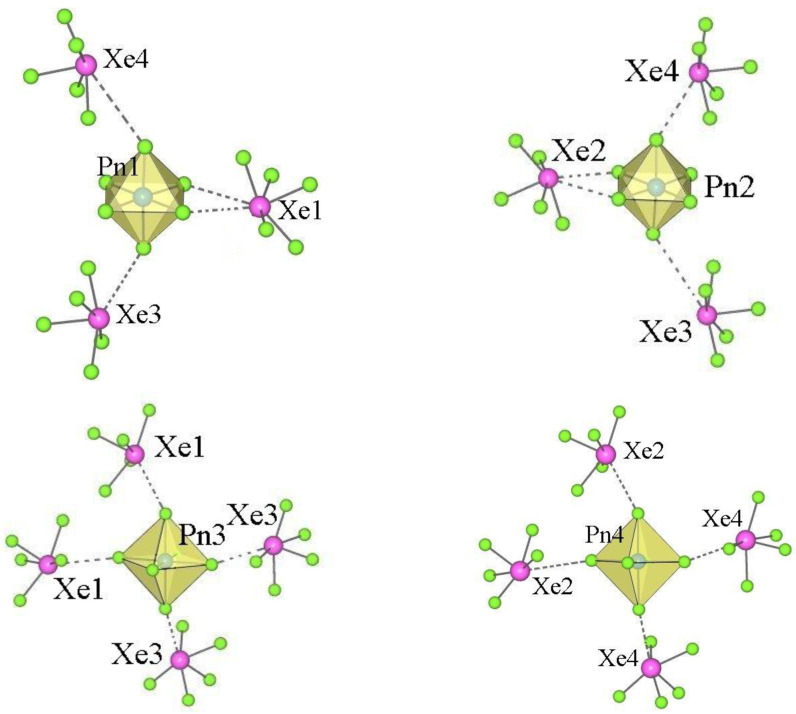
Secondary contacts between the [AF_6_]^−^ (A = As, Sb) anions and the surrounding [XeF_5_]^+^ cations in the crystal structure of *α*-[XeF_5_][As_0.5_Sb_0.5_F_6_].

In *α*-[XeF_5_][As_0.5_Sb_0.5_F_6_] [37], the cations [Xe(1)F_5_]^+^ and [Xe(2)F_5_]^+^ each form four secondary contacts (<3 Å) with the fluorine atoms of three AF_6_ groups (A = As, Sb), while [Xe(3)F_5_]^+^ and [Xe(4)F_5_]^+^ have three shorter (<3 Å) and one longer (3.437(7) Å and 3.420(7) Å, respectively) contact with four AF_6_ groups (Figure 9). Each of the crystallographically unique [A(1)F_6_]^−^ and [A(2)F_6_]^−^ anions forms four interactions with three [XeF_5_]^+^ cations, while the [A(3)F_6_]^−^ and [A(4)F_6_]^−^ anions form four interactions with four [XeF_5_]^+^ cations, (Figure 10).

#### 3.1.5. General Considerations for XeF_5_AF_6_ Salts (A = Nb, Ta, Ru, Rh, Os, Ir, Pt, Au, As, Sb)

**Figure 11:**
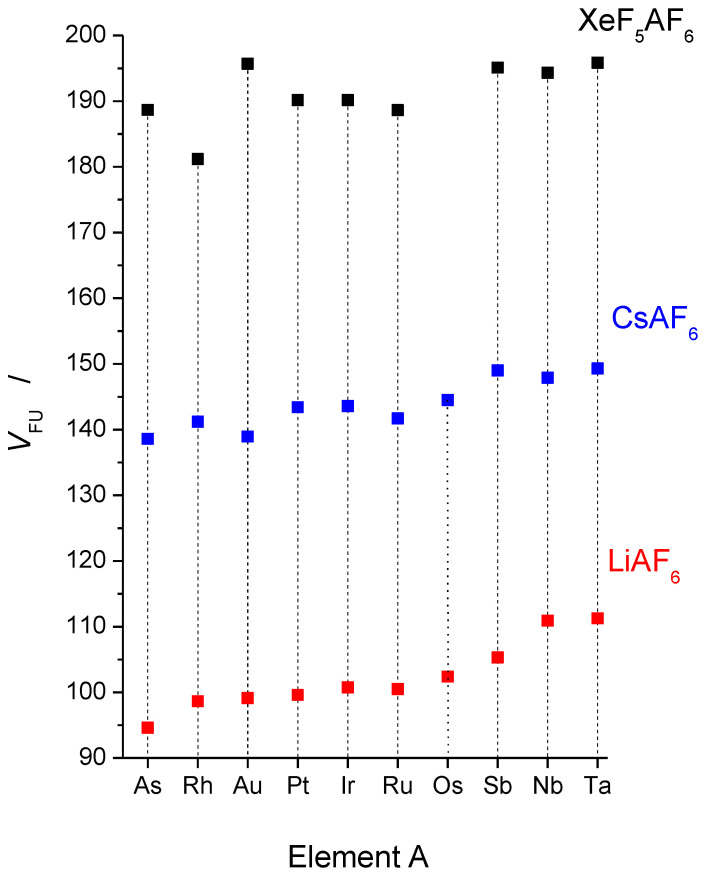
Formula unit volumes of LiAF_6_ (for A = Rh, Au, Pt, Ir, Ru, Os, Nb, Ta at 299 K and for A = As, Sb at room temperature), CsAF_6_ (all data at room temperature), and XeF_5_AF_6_ salts (all data at 150 K).

Table 9 lists the effective ionic radii *r*(A^5+^) (A = Nb, Ta, Ru, Rh, Os, Ir, Pt, Au, As, Sb) for coordination number six [56], the formula units (molecular) volumes *V*_FU_ of LiAF_6_, CsAF_6_, and XeF_5_AF_6_, and the average A–F bond lengths in LiAF_6_ and XeF_5_AF_6_. The molecular volumes of LiAF_6_, CsAF_6_, and XeF_5_AF_6_ are shown in Figure 11. The crystal structures of LiAF_6_ (A = Nb, Ta, Ru, Rh, Os, Ir, Pt, and Au) were determined by synchrotron X-ray powder diffraction at 299 K [57]. The crystal structures of LiAsF_6_ and LiSbF_6_ were determined by X-ray diffraction on powdered material and single crystals, respectively, at room temperature (RT) [58,59]. With the exception of CsAsF_6_ and CsSbF_6_, whose complete crystal structures were determined on single crystals at RT, only unit cells determined at RT are available for the other CsAF_6_ salts [60]. The crystal structures of CsAF_6_ (A = Rh, Pt, Ir, Os, and Au) were also determined on single crystals at 150 K [61]. The crystal structures of XeF_5_AF_6_ (A = Nb, Ta, Ru, Rh, Ir, Pt, Au, As [37], Sb [25]) were determined at 150 K and some of them also at RT (A = As [41], Au [39], Pt [24], Ru [38], Sb [37], Nb [30]).

Although the formula unit volumes *V*_FU_ of the LiAF_6_ and CsAF_6_ salts (Figure 11) show a similar trend, this is not the case for the XeF_5_AF_6_ salts, with the *V*_FU_ of XeF_5_RhF_6_ and XeF_5_AuF_6_ being particularly prominent. For LiAF_6_ and CsAF_6_, the *V*_FU_ are smallest for the As, Rh, and Au salts and largest for the Sb, Nb, and Ta salts. For the [XeF_5_]^+^ salts, the *V*_FU_ of XeF_5_AuF_6_ is almost identical to the *V*_FU_ of XeF_5_TaF_6_, while XeF_5_RhF_6_ has the smallest value *V*_FU_ of all XeF_5_AF_6_ salts (Table 9, Figure 11).

### 3.2. Crystal Structures of XeF_5_A_2_F_11_ (A = Nb, Ta, Sb)

**Figure 12:**
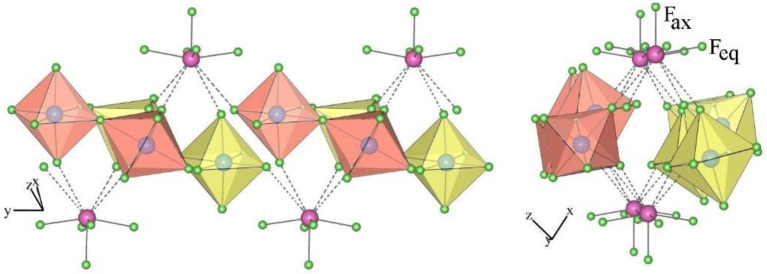
Secondary contacts between the [XeF_5_]^+^ cations and the [Sb_2_F_11_]^−^ anions in the crystal structure of XeF_5_Sb_2_F_11_.

**Figure 13:**
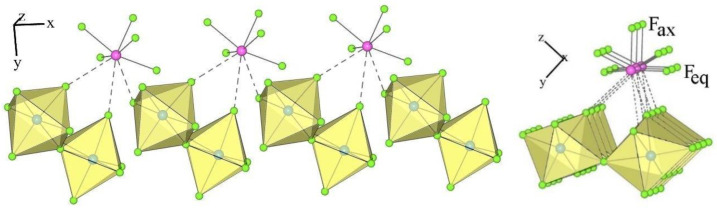
Secondary contacts between the [XeF_5_]^+^ cations and the [Nb_2_F_11_]^−^ anions in the crystal structure of XeF_5_Nb_2_F_11_.

**Figure 14:**
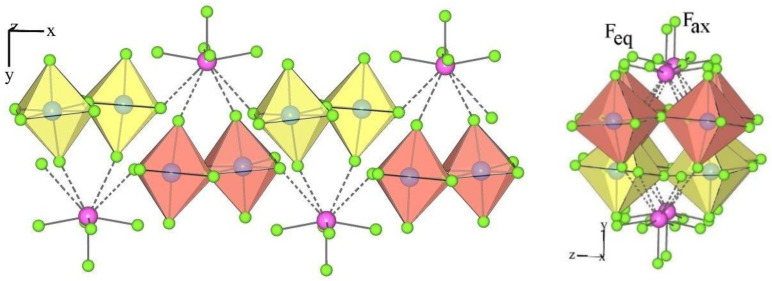
Secondary contacts between the [XeF_5_]^+^ cations and the [Ta_2_F_11_]^−^ anions in the crystal structure of XeF_5_Ta_2_F_11_.

In the XeF_6_–AF_5_ (A = Nb, Ta) system, only the salts XeF_5_AF_6_ and Xe_2_F_11_AF_6_ have been known so far [30,62,63]. The salts XeF_5_Nb_2_F_11_ and XeF_5_Ta_2_F_11_ were prepared for the first time in this study. As in the case of XeF_5_Sb_2_F_11_ [25] (Figure 12) the crystal structures of XeF_5_A_2_F_11_ (A = Nb, Ta) consist of discrete [XeF_5_]^+^ cations and dimeric [A_2_F_11_]^−^ anions interacting through secondary fluorine bridge Xe⋅⋅⋅F–A contacts (Figure 13 and Figure 14). Each crystal structure of the XeF_5_A_2_F_11_ salts (A = Sb, Nb, Ta) represents a unique example (Table 2 and Table 5).

In XeF_5_Nb_2_F_11_, each [XeF_5_]^+^ cation forms three secondary contacts with the fluorine atoms of two Nb_2_F_11_ groups, whereas in XeF_5_Sb_2_F_11_ and XeF_5_Ta_2_F_11_, each [A_2_F_11_]^−^ anion (A = Nb, Ta) participates in four secondary contacts with three different A_2_F_11_ dimers (Figure 12, Figure 13 and Figure 14). In all three salts, the Xe–F_ax_ bonds are shorter than the other four Xe–F_eq_ distances (Table 5). The A–F_b_–A bridge (Table 5) in the dimeric [A_2_F_11_]^−^ anion (A = Sb, Nb, Ta) is not linear as in [A_2_F_11_]^−^ (M = Nb, Ta) salts of protonated 1,3-dimethoxybenzene but bent as in many other examples [64]. In XeF_5_Sb_2_F_11_, the four F_eq_ atoms of the Sb(1)F_6_ unit are in staggered position in respect to the four F_eq_ of the Sb(2)F_6_ group with a torsion angle of ~37.5 ^o^, while in the corresponding Nb and Ta salts they are almost in an eclipsed position.

### 3.3. Crystal Structure of XeF_5_Ni(AsF_6_)_3_

**Figure 15:**
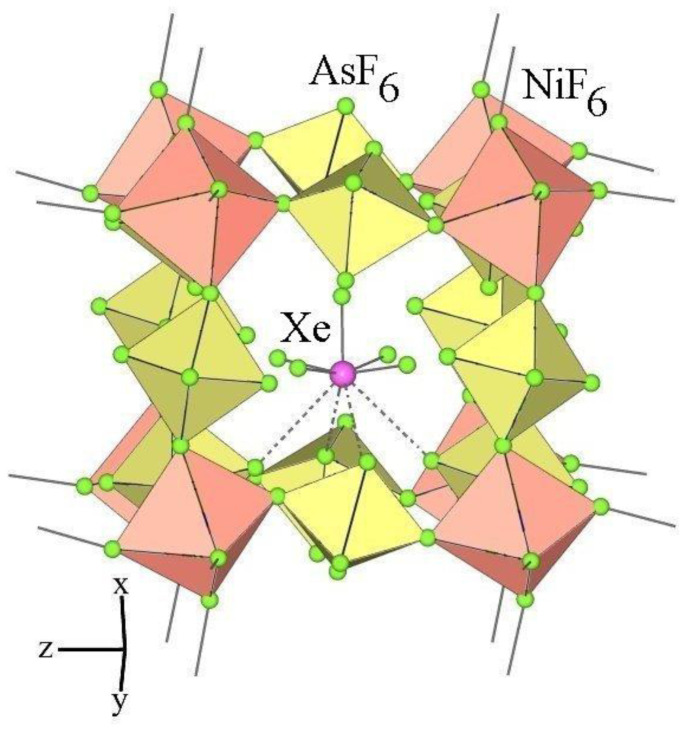
Part of the crystal structure of XeF_5_Ni(AsF_6_)_3_.

The crystal structure of XeF_5_Ni(AsF_6_)_3_ (Figure 15) is isotypical to the crystal structure of XeF_5_Ni(SbF_6_)_3_ [35]. The cation Ni^2+^ is coordinated by six fluorine atoms provided by six octahedral anions [AsF_6_]^−^ forming almost regular NiF_6_ octahedra. The Ni−F bond lengths in both salts are virtually identical (Table 6). They range from 1.989 (1) to 2.013 (1) Å. Due to the sharing of fluorine atoms, the NiF_6_ and AsF_6_ octahedra are connected to form a three-dimensional framework. The [XeF_5_]^+^ cations are located inside the cavities. The geometry of the [XeF_5_]^+^ cations is almost identical in both Ni salts (Table 5).

### 3.4. Crystal Structures of the Salts (Xe_2_F_11_)_2_(NiF_6_)_2_ and Ni(XeF_2_)_2_(IrF_6_)_2_

The crystal structure of (Xe_2_F_11_)_2_(NiF_6_)_2_ determined at 150 K is the same as that at room temperature [40], which means that there is no phase transition in the 150–296 K range.

It has been reported that the reaction between M^n+^(AF_6_)_n_^−^ and XeF_2_ in anhydrous aHF (aHF) leads to coordination compounds [M^n+^(XeF_2_)_p_](AF_6_)_n_^−^ (where XeF_2_ is coordinated to a metal cation M^n+^) only when the Lewis acidity of M^n+^ is not high enough to withdraw F^−^ ions from XeF_2_ to form MF_n_ and Xe_2_F_3_^+^AF_6_^−^ [65]. Since the reaction between Ni(AsF_6_)_2_ and XeF_2_ in aHF gave NiF_2_ and Xe_2_F_3_AsF_6_, the preparation of [Ni(XeF_2_)_2_](IrF_6_)_2_ was a small surprise.

**Figure 16:**
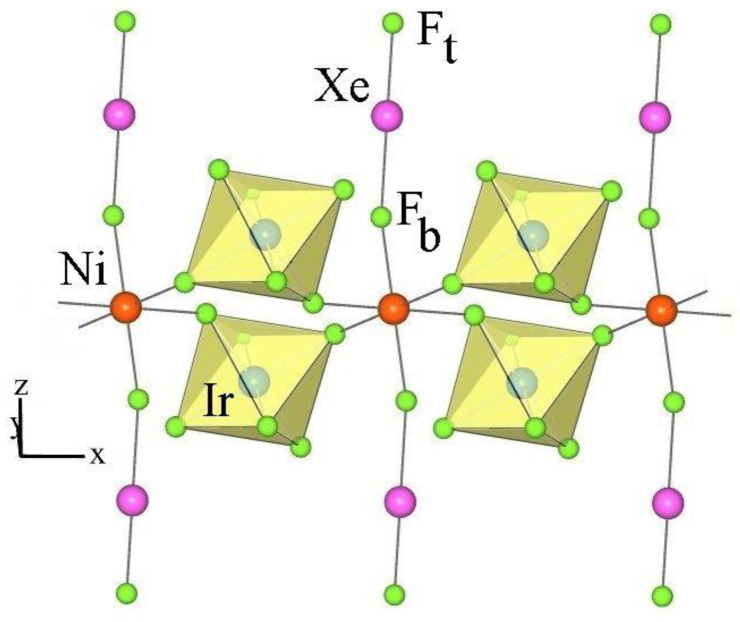
Part of the crystal structure of [Ni(XeF_2_)_2_](IrF_6_)_2_.

The crystal structure of [Ni(XeF_2_)_2_](IrF_6_)_2_ is isotypical to the crystal structure of [Cu(XeF_2_)_2_](SbF_6_)_2_ [54]. In both structures, adjacent [M(XeF_2_)_2_]^2+^ units are connected via two [AF_6_]^−^ units with bridging fluorine atoms in the cis position to form infinite chains that are parallel to the *x*–axis (Figure 16). These chains are interconnected by weak F_2_Xe···F–AF_5_ contacts and form a three-dimensional network.

The metal cation Ni^2+^ is sixfold coordinated by fluorine atoms of two XeF_2_ ligands and four [IrF_6_]^−^ anions. In [Cu(XeF_2_)_2_](SbF_6_)_2_, the Cu^2+^ cation is coordinated by two XeF_2_ molecules (Cu–F = 2 × 1.857 (5) Å) and four fluorine atoms provided by four [SbF_6_] units with two shorter and two longer Cu–F bonds (2 × 2.090 (5) Å and 2 × 2.123 (5) Å) [54]. In contrast, the Ni–F(Ir) bonds in the Ni(XeF_2_)_2_(IrF_6_)_2_ salt are almost the same length (2 × 2.016 (9) Å and 2 × 2.023 (7) Å). The Ni–F(XeF_2_) bonds are much longer (2 × 1.938(6) Å) than in the [Cu(XeF_2_)_2_](SbF_6_)_2_ salt (2 × 1.857 (5) Å), indicating a weaker M^2+^–FXeF interaction in the Ni salt. Consequently, Xe–F_b_ (F_b_ = bridging F atom) is shorter (2.078 (6) Å) in the Ni salt than in the Cu salt (2.102 (5) Å) and the opposite is true for Xe−F_t_ bonds [F_t_ = terminal fluorine atom; 1.920 (7) Å (Ni salt) and 1.906 (5) Å (Cu salt)].

## 4. Materials and Methods

CAUTION: Anhydrous HF and some fluorides are highly toxic and must be handled under a well-ventilated hood, and protective clothing must be worn at all times*!*

### 4.1. Apparatus, Techniques, and Reagents

The handling of volatile (anhydrous HF, F_2_, AsF_5_, BF_3_) and nonvolatile materials and the type of the reaction and crystallization vessels have already been reported [35,47,66]. Metallic Re powder (Alfa Aesar, Haverhill, MA, USA, 99.99%), Ru sponge (Alfa Aesar, 99.95%), Rh sponge (BDH, 99.9%), Os powder (Alfa Aesar, 99.8%), Ir sponge (Alfa Aesar, 99.95%), Pt powder (Aldrich, St. Louis, MO, USA, 99.9%), elemental F_2_ (Solvay Fluor and Derivate GmbH, Hannover, Germany 99.98%), CrF_3_ (Messer Griesheim, Bad Soden, Germany, 99.9%), and BF_3_ (Union Carbide Austria, GmbH, 99.5%) were used as supplied. Anhydrous HF (Linde AG, Pullach, Germany, 99.995%) was treated with K_2_NiF_6_ (Advance Research Chemicals Inc, Catoosa, OK, USA, 99.9%) for several hours before use. NiF_2_ (Alfa Products, Bedford Park, IL, USA, 99.5%) and CuF_2_ (Aldrich, 98%) were treated with elemental F_2_ at 220 °C for several hours before use. NbF_5_ (Alfa Aesar, 99%) and TaF_5_ (Alfa Aesar, 99.9%) were sublimed before use. AuF_3_ [66], XeF_2_ [67], AsF_5_ [68], XeF_5_SbF_6_ [34], Sn(SbF_6_)_2_ [69,70], Pb(SbF_6_)_2_ [69,70], and Zn(SbF_6_)_2_ [69,70] were synthesized as described previously.

Raman spectra were recorded at room temperature using a Renishaw Raman Imaging Microscope System 1000 or a Horiba Jobin Yvon LabRam-HR spectrometer [66].

### 4.2. Attempted Preparation of XeF_5_M(AF_6_)_3_ (M = Cu, Ni; A = Cr, Nb, Ta, Ru, Rh, Re, Os, Ir, Pt, Au, As), XeF_5_M(SbF_6_)_3_ (M = Sn, Pb), and XeF_5_M(BF_4_)_x_(SbF_6_)_3-x_ (x = 1, 2, 3; M = Co, Mn, Ni, Zn)

The solid starting reagents were loaded into reaction vessels in a dry box (Table S1). The solvent HF and optionally BF_3_, AsF_5_, and SbF_5_ were condensed at 77 K to solid reagents, and the reaction vessel was warmed to ambient temperature. Fluorine was slowly added to the reaction vessel at room temperature. A medium-pressure mercury lamp (Hg arc lamp, 450 W, Ace Glass, Vineland, NJ, USA) was used as the UV source. After several days of intensive stirring at room temperature, the volatiles were pumped off and the Raman spectra of the obtained solids were recorded (Figures S1–S11).

For crystallization, the clear supernatant, which contained no visible sediment, was decanted into the side arm of the crystallization vessel, which consisted of two tubes made of fluoropolymer. Evaporation of the solvent from the side arm was achieved by maintaining a temperature gradient of about 10–20 °C between the two tubes for several weeks. Slow distillation of aHF resulted in crystal growth.

Crystals were immersed in perfluorodecalin (melting point 263 K) in a dry box, selected under the microscope, and mounted on the goniometer head of the diffractometer in a cold nitrogen stream (265–273 K). Some of them were sealed in quartz capillaries used to record Raman spectra at several random positions (Figures S1–S11).

### 4.3. Crystal Structure Determination

Single-crystal X-ray diffraction data of reported crystal structures were acquired at 150 K (for XeF_5_IrF_6_ also at 285 K) with a Gemini A diffractometer equipped with an Atlas CCD detector using graphite monochromated MoKα radiation. The data were processed using the CrysAlisPro software suite program package [71]. Analytical absorption corrections were applied to all data sets. All structures were solved using the dual-space algorithm of the program SHELXT [72] implemented in the Olex crystallographic software [73]. Structure refinement for all structures was performed using the software SHELXL-2014 [74]. The crystals of the Ni(XeF_2_)_2_(IrF_6_)_2_ salt showed reproducible pseudo-merohedral twinning. This problem was solved at the data processing stage, and final refinement was performed using reflections from the main domain. The figures were created using the software Balls and Sticks [75]. The compound XeF_5_Nb_2_F_11_ crystallizes in the acentric space group *P2_1_*. The very-close-to-zero value of the Flack’s parameter (−0.031 (11)) confirms the correctness of the absolute structure.

The supplementary crystallographic data for this work are provided free of charge by the joint Cambridge Crystallographic Data Centre and the Fachinformationszentrum Karlsruhe Access Structures service www.ccdc.cam.ac.uk/structures (accessed on March 10, 2023): CSD-2246135 [(Xe_2_F_11_)_2_NiF_6_], CSD-2246136 [Ni(XeF_2_)_2_(IrF_6_)_2_], CSD-2246137 [XeF_5_AuF_6_], CSD-2246138 [XeF_5_IrF_6_, 150 K], CSD-2246139 [XeF_5_Nb_2_F_11_], CSD-2246140 [XeF_5_PtF_6_], CSD-2246141 [XeF_5_RuF_6_], CSD-2246142 [XeF_5_IrF_6_, 280 K], CSD-2246143 [XeF_5_Ta_2_F_11_], CSD-2246144 [XeF_5_NbF_6_], CSD-2246145 [XeF_5_Ni(AsF_6_)_3_], CSD-2246146 [XeF_5_TaF_6_], CSD-2246147 [XeF_5_RhF_6_].

## 5. Conclusions

Although the experiments to prepare XeF_5_M(BF_4_)_x_(SbF_6_)_3-x_ (x = 1, 2, 3; M = Co, Mn, Ni, Zn), XeF_5_M(SbF_6_)_3_ (M = Sn, Pb), and XeF_5_M(AF_6_)_3_ salts (M = Cu, Ni; A = Cr, Nb, Ta, Ru, Rh, Re, Os, Ir, Pt, Au, As) were successful only in the preparation of XeF_5_Ni(AsF_6_)_3_, further valuable results were obtained:

(a) In view of the successful preparation of XeF_5_Ni(AsF_6_)_3_, we assume that it is reasonable to attempt the preparation of other compounds with other M^2+^ cations (M = Mg, Fe, Co, Zn, etc.).
(b) Crystal structure determination of XeF_5_RhF_6_ reveals a new type of structure. Together with the crystal structures of XeF_5_TaF_6_ and XeF_5_IrF_6_, which were determined for the first time, and the redetermined crystal structures of XeF_5_NbF_6_, XeF_5_PtF_6_, XeF_5_RuF_6_, and XeF_5_AuF_6_, they contribute to the understanding of the possible crystal phases in the family of XeF_5_AF_5_ salts.
(c) The crystal structures of the XeF_5_Nb_2_F_11_ and XeF_5_Ta_2_F_11_ salts were determined. These compounds were previously unknown, and for the XeF_5_A_2_F_11_ salts, only the crystal structure of XeF_5_Sb_2_F_11_ [25] was known. These three [A_2_F_11_]^−^ salts are not isotypic and each of them represents a unique structural type.
(d) The crystal structure of XeF_5_IrF_6_ determined at 150 K and at room temperature is identical. The crystal structures of the salts XeF_5_NbF_6_, XeF_5_PtF_6_, XeF_5_RuF_6_, XeF_5_AuF_6_, XeF_5_AsF_6_ [37], and (Xe_2_F_11_)_2_NiF_6_ redetermined at 150 K are also identical to those determined at room temperature, indicating that there is no phase transition in the range from 150 K to 298 K.
(e) All the new data on the XeF_5_AF_6_ and XeF_5_A_2_F_11_ salts help to fill the gaps in our knowledge of the XeF_6_-A^V^F_5_ system (Table 10).
(f) The preparation of Ni(XeF_2_)_2_(IrF_6_)_2_ has shown that it is worthwhile to try the preparation of some other [M^n+^(XeF_2_)_p_](AF_6_)_n_^−^ salts (A = Rh, Ru, Os, Ir, Pt, Au, Nb, Ta) where attempts to stabilize such salts with [AF_6_]^−^ (A = As, Sb) have failed.

## Figures and Tables

**Table 1:** Summary of crystal data and refinement results for XeF_5_AF_6_ (A = Nb, Ta, Ru, Rh, Ir, Pt, Au).

Formula	XeF_5_NbF_6_	XeF_5_TaF_6_	XeF_5_RuF_6_	XeF_5_RhF_6_
*T* (K)	150	150	150	150
Crystal System	Orthorhombic	Orthorhombic	Orthorhombic	Orthorhombic
Space Group	*Pnma*	*Pnma*	*Pnma*	*Pbca*
*a* (Å)	16.8078 (10)	16.8312 (12)	16.6197 (12)	9.0028 (4)
*b* (Å)	8.2491 (6)	8.2399 (6)	8.0530 (6)	8.8181 (4)
*c* (Å)	5.6064 (3)	5.6488 (4)	5.6373 (4)	18.2581 (8)
*V* (Å^3^)	777.32 (8)	783.41 (10)	754.49 (9)	1449.47 (11)
*Z*	4	4	4	8
*D*_calcd_ (g/cm^3^)	3.702	4.419	3.886	4.062
*λ* (Å)	0.71073	0.71073	0.71073	0.71073
*μ* (mm^−1^)	5.988	18.436	6.652	7.118
GOF ^a^	1.091	1.052	1.109	1.044
*R*_1_ ^b^	0.0253	0.0355	0.0244	0.0226
*wR*_2_ ^c^	0.0503	0.0757	0.0521	0.0430
**Formula**	**XeF_5_IrF_6_**	**XeF_5_IrF_6_**	**XeF_5_PtF_6_**	**XeF_5_AuF_6_**
***T*** **(K)**	**150**	**285**	**150**	**150**
**Crystal System**	**Orthorhombic**	**Orthorhombic**	**Orthorhombic**	**Monoclinic**
**Space Group**	***Pnma***	***Pnma***	***Pnma***	***P2_1_/c***
*a* (Å)	16.5720 (10)	16.7456 (14)	16.5286 (13)	5.8447 (5)
*b* (Å)	7.9954 (5)	8.1444 (8)	7.9642 (5)	16.6324 (10)
*c* (Å)	5.7412 (4)	5.6998 (6)	5.7779 (5)	8.0536 (5)
*β (^o^)*				90.781 (6)
*V* (Å^3^)	760.71 (8)	777.35 (13)	760.58 (10)	782.82 (9)
*Z*	4	4	4	4
*D*_calcd_ (g/cm^3^)	4.650	4.550	4.675	4.559
*λ* (Å)	0.71073	0.71073	0.71073	0.71073
*μ* (mm^−1^)	22.088	21.615	22.986	23.202
GOF ^a^	1.081	1.174	1.078	1.064
*R*_1_ ^b^	0.0267	0.0398	0.0247	0.0364
*wR*_2_ ^c^	0.0491	0.0815	0.0445	0.0798

^a^ GOF = [Σ*w*(*F*_o_^2^ − *F*_c_^2^)^2^/(*N*_o_ − *N*_p_)]^1/2^, where *N*_o_ = no. of reflns and *N*_p_ = no. of refined parameters. ^b^ *R*_1_ = Σ׀׀*F*_o_׀ − ׀*F*_c_׀׀/Σ׀*F*_o_׀. ^c^ *wR*_2_ = [Σ*w*(*F*_o_^2^ − *F*_c_^2^)^2^/Σ(*w*(*F*_o_^2^)^2^]^1/2^.

**Table 2:** Summary of crystal data and refinement results for XeF_5_A_2_F_11_ (A = Nb, Ta), XeF_5_Ni(AsF_6_)_3_, (Xe_2_F_11_)_2_(NiF_6_)_2_, and Ni(XeF_2_)_2_(IrF_6_)_2_.

Formula	XeF_5_Nb_2_F_11_	XeF_5_Ta_2_F_11_	XeF_5_Ni(AsF_6_)_3_	(Xe_2_F_11_)_2_(NiF_6_)_2_	Ni(XeF_2_)_2_(IrF_6_)_2_
*T* (K)	150	150	150	150	150
Crystal System	Monoclinic	Monoclinic	Monoclinic	Monoclinic	Monoclinic
Space Group	*P2_1_*	*I2/a*	*P2_1_/n*	*I2/c*	*P2_1_/c*
*a* (Å)	5.2717 (2)	8.9972 (5)	10.2200 (3)	17.2498 (11)	5.43790 (10)
*b* (Å)	14.1920 (5)	9.3302 (5)	10.1973 (3)	5.3239 (3)	14.6396 (5)
*c* (Å)	7.6489 (2)	14.0691 (8)	14.5606 (4)	21.0164 (11)	9.1039 (3)
*β (^o^)*	92.412 (3)	99.281 (5)	90.072 (2)	102.510 (6)	92.003 (2)
*V* (Å^3^)	571.75 (3)	1165.58 (11)	1517.45 (8)	1884.3 (2)	724.31 (4)
*Z*	2	4	4	4	2
*D*_calcd_ (g/cm^3^)	3.608	4.543	3.728	3.934	4.630
*λ* (Å)	0.71073	0.71073	0.71073	0.71073	0.71073
*μ* (mm^−1^)	5.115	21.814	10.216	8.358	24.375
GOF ^a^	1.056	1.057	1.062	1.045	1.072
*R*_1_ ^b^	0.0205	0.0273	0.0269	0.0214	0.0429
*wR*_2_ ^c^	0.0427	0.0665	0.0563	0.0463	0.1305

^a^ GOF = [Σ*w*(*F*_o_^2^ − *F*_c_^2^)^2^/(*N*_o_ − *N*_p_)]^1/2^, where *N*_o_ = no. of reflns and *N*_p_ = no. of refined parameters. ^b^ *R*_1_ = Σ׀׀*F*_o_׀ − ׀*F*_c_׀׀/Σ׀*F*_o_׀. ^c^ *wR*_2_ = [Σ*w*(*F*_o_^2^ − *F*_c_^2^)^2^/Σ(*w*(*F*_o_^2^)^2^]^1/2^.

**Table 3:** Geometric parameters (Å) of the [XeF_5_]^+^ cations, secondary Xe···F contacts (Å), and A–F bond lengths (Å) in the crystal structures of isotypic XeF_5_AF_6_ (A = Nb, Ta, Ru, Ir, Pt, Au) and literature data for XeF_5_SbF_6_.

	Nb ^a^	Ta ^a^	Ru ^a^	Ir ^a^	Pt ^a^	Sb ^b^
Orthorhombic *Pnma*						
	150 K	150 K	150 K	150 K	150 K	150 K
Xe–F_ax_	1.808 (3)	1.803 (7)	1.807 (3)	1.809 (5)	1.809 (5)	1.804 (3)
Xe–F_eq_	1.839 (2)	1.839 (5)	1.843 (2)	1.835 (3)	1.838 (3)	1.841 (2)
	1.839 (2)	1.839 (5)	1.843 (2)	1.835 (3)	1.838 (3)	1.841 (2)
	1.842 (2)	1.843 (5)	1.846 (2)	1.843 (3)	1.839 (3)	1.844 (2)
	1.842 (2)	1.843 (5)	1.846 (2)	1.843 (3)	1.839 (3)	1.844 (2)
Xe···F	2.540 (3)	2.562 (6)	2.562 (3)	2.602 (5)	2.604 (5)	2.617 (3)
	2.535 (2)	2.560 (6)	2.587 (3)	2.616 (4)	2.646 (4)	2.638 (3)
	2.887 (2)	2.894 (5)	2.856 (2)	2.822 (3)	2.817 (3)	2.860 (2)
	2.887 (2)	2.894 (5)	2.857 (3)	2.822 (3)	2.817 (3)	2.860 (2)
A–F	1.835 (3)	1.849 (7)	1.820 (3)	1.841 (5)	1.857 (4)	1.857 (3)
	1.852 (3)	1.873 (7)	1.823 (3)	1.861 (5)	1.871 (4)	1.859 (3)
	1.886 (2)	1.880 (5)	1.854 (3)	1.879 (3)	1.884 (3)	1.882 (2)
	1.886 (2)	1.880 (5)	1.854 (3)	1.879 (3)	1.884 (3)	1.882 (2)
	1.925 (3)	1.930 (6)	1.878 (3)	1.892 (4)	1.894 (4)	1.895 (3)
	1.941 (3)	1.939 (6)	1.885 (3)	1.906 (5)	1.897 (4)	1.898 (3)

^a^ This work. ^b^ Ref. [25].

**Table 4:** Geometric parameters (Å) of the [XeF_5_]^+^ cations, secondary Xe···F contacts (Å), and A–F bond lengths (Å) in the crystal structures of XeF_5_AF_6_ (A = Rh, Au) and literature data for XeF_5_AsF_6_ (monoclinic and orthorhombic phase).

	Rh ^a^	Au ^a^	As ^b^	As ^c^
	Orthorhombic *Pbca*	Monoclinic *P*2_1_/*c*	Monoclinic *P*2_1_/*c*	Orthorhombic *Ama2*
	150 K	150 K	150 K	100 K
				Xe (2)/Xe (1)
Xe–F_ax_	1.814 (2)	1.804 (5)	1.794 (3)	1.815 (7)/1.800 (8)
Xe–F_eq_	1.833 (2)	1.823 (6)	1.826 (3)	1.844 (5)/1.840 (6)
	1.841 (2)	1.834 (6)	1.828 (3)	1.833 (8)/1.838 (6)
	1.842 (2)	1.835 (5)	1.833 (3)	1.833 (8)/1.838 (6)
	1.842 (2)	1.836 (5)	1.836 (3)	1.832 (8)/1.840 (6)
Xe···F	2.603 (2)	2.575 (6)	2.643 (3)	2.705 (5)/2.615 (6)
	2.664 (2)	2.746 (5)	2.722 (3)	2.705 (5)/2.615 (6)
	2.761 (2)	2.785 (6)	2.782 (3)	2.767 (6)/2.796 (6)
	2.844 (2)			2.767 (6)/3.451 (7)
				As (2)/As (1)
A–F	1.871 (2)	1.872 (6)	1.688 (3)	1.709 (5)/1.657 (7)
	1.873 (2)	1.876 (5)	1.696 (3)	1.709 (5)/1.657 (7)
	1.874 (2)	1.884 (5)	1.696 (3)	1.741 (6)/1.699 (6)
	1.879 (2)	1.903 (5)	1.738 (3)	1.749 (5)/1.699 (6)
	1.799 (2)	1.910 (5)	1.744 (3)	1.749 (5)/1.712 (6)
	1.815 (2)	1.911 (5)	1.750 (3)	1.710 (7)/1.712 (6)

^a^ This work. ^b^ Ref. [37]. ^c^ Ref. [53].

**Table 5:** Geometric parameters (Å) of the [XeF_5_]^+^ cations, secondary Xe···F contacts (Å), and A–F (A = Nb, Ta) bond lengths (Å) and A–F_b_–A angles (^o^) in the crystal structures of XeF_5_A_2_F_11_ (A = Nb, Ta) and literature data for XeF_5_Sb_2_F_11_.

	Nb ^a^	Ta ^a^	Sb ^b^
	Monoclinic *P*2_1_	Monoclinic *I*2/*a*	Triclinic *P* $\bar{1}$
	150 K	150 K	200 K
Xe–F_ax_	1.801 (3)	1.802 (6)	1.883 (3)
Xe–F_eq_	1.826 (3)	1.838 (3)	2.019 (3)
	1.836 (3)	1.838 (3)	2.029 (3)
	1.837 (3)	1.839 (4)	1.837 (3)
	1.838 (4)	1.839 (4)	1.838 (3)
Xe···F	2.582 (3)	2.666 (4)	2.915 (3)
	2.633 (3)	2.872 (3)	2.848 (3)
	2.667 (3)	2.872 (3)	2.775 (3)
			2.814 (3)
A (1)–F_t_	1.823 (3)	1.824 (4)	1.838 (3)
	1.840 (4)	1.849 (4)	1.846 (3)
	1.846 (4)	1.855 (4)	1.853 (3)
	1.847 (4)	1.900 (4)	1.866 (3)
	1.918 (3)	1.910 (4)	1.883 (3)
A (1)–F_b_	2.096 (3)	2.0657 (6)	2.019 (3)
		2.0657 (6)	
A (2)–F_t_	1.821 (4)		1.837 (3)
	1.832 (3)		1.838 (3)
	1.833 (4)		1.843 (3)
	1.910 (3)		1.870 (3)
	1.923 (3)		1.878 (3)
A (2)–F_b_	2.038 (3)		2.029 (3)
A–F_b_–A	155.63 (19)	169.6 (3)	145.09 (16)

^a^ This work. ^b^ Ref. [25].

**Table 6:** Experimental geometric parameters (Å) of the two crystallographically independent Ni^+^ cations, geometric parameters (Å) of the [XeF_5_]^+^ cations, and secondary Xe···F contacts in the crystal structures of XeF_5_Ni(AsF_6_)_3_ and literature data for XeF_5_Ni(SbF_6_)_3_.

	XeF_5_Ni (AsF_6_)_3_^a^	XeF_5_Ni (SbF_6_)_3_^b^
	*P*2_1_/*n*	
	150 K	
Ni (1)–F	2.000 (2)	2.002 (1)
	2.000 (2)	2.002 (1)
	1.994 (2)	1.989 (1)
	1.994 (2)	1.989 (1)
	2.006 (2)	2.013 (1)
	2.006 (2)	2.013 (1)
Ni (2)–F	1.999 (2)	1.991 (1)
	1.999 (2)	1.991 (1)
	2.006 (2)	1.979 (1)
	2.006 (2)	1.979 (1)
	2.010 (2)	2.006 (1)
	2.010 (2)	2.006 (1)
Xe–F_ax_	1.782 (2)	1.800 (2)
Xe–F_eq_	1.818 (2)	1.825 (2)
	1.819 (2)	1.828 (2)
	1.820 (2)	1.826 (2)
	1.822 (2)	1.832 (2)
Xe···F	2.903 (2)	2.866 (2)
	2.919 (3)	2.928 (2)
	2.931 (2)	2.944 (2)
	2.971 (3)	2.898 (2)

^a^ This work. ^b^ Ref. [35].

**Table 7:** Experimental geometric parameters (Å) in the crystal structure of [Ni(XeF_2_)_2_](IrF_6_)_2_ and literature data for [Cu(XeF_2_)_2_](SbF_6_)_2_.

	[Ni (XeF_2_)_2_] (IrF_6_)_2_^a^	[Cu (XeF_2_)_2_] (SbF_6_)_2_^b^
	*P*2_1_/*c*	
	150 K	200 K
M–F_b_ (AF_6_)	2.016 (6)	2.090 (5)
	2.016 (6)	2.090 (5)
	2.023 (7)	2.123 (5)
	2.023 (7)	2.123 (5)
M–F_b_ (XeF_2_)	1.938 (6)	1.857 (5)
	1.938 (6)	1.857 (5)
Xe–F_t_	1.920 (7)	1.906 (5)
Xe–F_b_	2.078 (6)	2.102 (5)
A–F_b_	1.921 (7)	1.891 (5)
	1.934 (7)	1.917 (5)
A–F_t_	1.843 (8)	1.841 (6)
	1.852 (8)	1.843 (6)
	1.858 (8)	1.861 (6)
	1.861 (8)	1.870 (6)

^a^ This work. ^b^ Ref. [54].

**Table 8:** Summary of crystal data for the orthorhombic XeF_5_AsF_6_ and mixed anion [XeF_5_][As_0.3_Sb_0.7_F_6_) and [XeF_5_][As_0.5_Sb_0.5_F_6_) salts.

Compound	Crystal System	Space Group	*Z*	*a* /Å	*b* /Å	*c* /Å	*T* /K
XeF_5_AsF_6_^a^	orthorhombic	*Ama*2	8	9.796 (2)	13.272 (10)	11.578 (2)	100
[XeF_5_][As_0.3_Sb_0.7_F_6_] ^b^	orthorhombic	*Ama2(00γ)s0s*	8	10.031 (1)	13.362 (1)	11.808 (1)	200
*β*-[XeF_5_][As_0.5_Sb_0.5_F_6_] ^b^	orthorhombic	*Ama2(00γ)s0s*	8	10.1196 (5)	13.4517 (6)	11.8999 (5)	295
*α*-[XeF_5_][As_0.5_Sb_0.5_F_6_] ^b^	orthorhombic	*Pca2_1_*	16	9.9738 (2)	13.2492 (4)	23.3701 (7)	150

^a^ Ref. [53]. ^b^ Ref. [37].

**Table 9:** Effective ionic radii *r*(A^5+^) (A = Nb, Ta, Ru, Rh, Os, Ir, Pt, Au, As, Sb) for coordination number six (Å), formula unit (molecular) volumes *V*_FU_ (Å^3^) of LiAF_6_, CsAF_6_, and XeF_5_AF_6_, and average A–F bond lengths *d*_av_[A–F] (Å) in LiAF_6_ and XeF_5_AF_6_.

A	As	Rh	Au	Pt	Ir	Ru	Os	Sb	Nb	Ta
*r*(A^5+^) ^a^	0.46	0.55	0.57	0.57	0.57	0.565	0.575	0.60	0.64	0.64
**LiAF_6_**										
*V*_FU_	94.6 ^b^	98.64 ^c^	99.12 ^c^	99.61 ^c^	100.77 ^c^	100.5 ^c^	102.41 ^c^	105.3 ^d^	110.92 ^c^	111.26 ^c^
*d*_av_[A–F]	1.74	1.855	1.874	1.887	1.879	1.851	1.872	1.877	1.863	1.859
**CsAF_6_**										
*V*_FU_ ^e^	138.6	141.2	138.95	143.4	143.6	141.7	144.5	149	147.9	149.3
*V*_FU_ ^f^		137.07		138.42	138.97	138.35	139.97			
**XeF_5_AF_6_**										
Type	II	III	II	I	I	I	I	I	I	I
*V*_FU_ ^g^	196.15		203.03	196.35	194.34	193.26		199.42	200	
*V*_FU_ ^h^	188.67	181.18	195.71	190.15	190.18	188.62		195.11	194.33	195.85
*d*_av_[A–F]	1.719	1.852	1.893	1.881	1.876	1.852		1.879	1.888	1.892

^a^ Ref. [56]. ^b^ Room temperature (RT); Ref. [58]. ^c^ 299 K; Ref. [57]. ^d^ RT; Ref. [59]. ^e^ RT; Ref. [60]. ^f^ 150 K; Ref. [61]. ^g^ 285 K for Ir salt (this work); others RT (Refs. [24,30,37,38,39,41]). ^h^ This work (150 K) except XeF_5_AsF_6_ (150 K) [37] and XeF_5_SbF_6_ (150 K) [25].

**Table 10:** List of known xenon(VI) fluoridometallates including the determined crystal structures (letters in bold). Crystal structures reported in this work are highlighted in green.

Formula		A^5+^												
[Xe_2_F_11_][AF_6_]	**V** ^a^	Nb ^b^	Ta ^c^	Ru ^d^			Ir ^e^	Pt ^f^	**Au** ^d^	U ^g^	P ^h^	As ^i^	**Sb** ^j^	Bi ^k^
[XeF_5_][AF_6_]	V ^l^	**Nb** ^m^	**Ta** ^c^	**Ru** ^n^	**Rh**	Os ^o^	**Ir** ^e^	**Pt** ^p^	**Au** ^f^	U ^r^		**As** ^s^	**Sb** ^t^	Bi ^u^
[XeF_5_][A_2_F_11_]	V ^l^	**Nb**	**Ta**										**Sb** ^t^	

^a^ Refs. [21,27], ^b^ Ref. [62], ^c^ Ref. [63], ^d^ Refs. [26,39], ^e^ Refs. [23,39], ^f^ Ref. [39], ^g^ Ref. [76], ^h^ Ref. [22], ^i^ Refs. [22,29], ^j^ Refs. [19,28], ^k^ Ref. [77], ^l^ Ref. [78], ^m^ Refs. [30,62], ^n^ Ref. [38], ^o^ Ref. [39], ^p^ Ref. [24], ^r^ Ref. [79], ^s^ Refs. [20,22,41], ^t^ Refs. [19,25], ^u^ Ref. [80].

## Data Availability

Not applicable.

## Supplementary Materials

The following supporting information can be downloaded at: https://www.mdpi.com/article/10.3390/molecules28083370/s1, Table S1: Experimental conditions and observed products upon crystallization for the reactions between UV-irradiated F_2_, XeF_2_, MF_2_ (M = Cu, Ni) and metal A (A = Ru, Rh, Re, Os, Ir, Pt), MF_3_ (A = Cr, Au), and AF_5_ (M = Nb, Ta, As), respectively, in anhydrous HF. The products observed upon crystallization and the experimental conditions for the reactions between XeF_5_SbF_6_ and M(SbF_6_)_2_ (M = Sn, Pb) are also given.; Table S2: Experimental conditions and observed products upon crystallization in the experiments to prepare XeF_5_M(BF_4_)_x_(SbF_6_)_3-x_ (x = 1, 2, 3; M = Co, Mn, Ni, Zn) salts.; Figures S1–S10. Raman spectra of XeF_5_NbF_6_, XeF_5_TaF_6_, XeF_5_RhF_6_, XeF_5_RuF_6_, XeF_5_IrF_6_, XeF_5_PtF_6_, XeF_5_Nb_2_F_11_, XeF_5_Ta_2_F_11_, O_2_PtF_6_, and O_2_RuF_6_ recorded on a single crystal; Figure S11. Raman spectra of single crystals after crystallization of the reaction product between XeF_2_, Os powder and UV-irradiated F_2_ in anhydrous HF: XeF_4_ and unknown product.

## Sample Availability

Samples of the compounds are available from the authors.
