# Supplementary material for: Crystal Structures of Xenon(VI) Salts: XeF5Ni(AsF6)3, XeF5AF6 (A = Nb, Ta, Ru, Rh, Ir, Pt, Au), and XeF5A2F11 (A = Nb, Ta)
Source: Molecules. 2023 Apr 11;28(8):3370. doi: 10.3390/molecules28083370 (PMC10143524; doi:10.3390/molecules28083370)
Supplement: Supplementary file 1 [file molecules-28-03370-s001.zip › molecules-2324196-supplementary.pdf]

Crystal structures of xenon(VI) salts: $XeF_5Ni(AsF_6)_3$, XeF_5AF_6 (A = Nb, Ta, Ru, Rh, Ir, Pt, Au), and $XeF_5A_2F_{11}$ (A = Nb, Ta)

Zoran Mazej [1,*] and Evgeny Goreshnik [1]

[1] Department of Inorganic Chemistry and Technology, Jožef Stefan Institute; Jamova cesta 39, SI-1000 Ljubljana, Slovenia
* Correspondence: zoran.mazej@ijs.si

Table S1. Experimental conditions and observed products upon crystallization for the reactions between UV-irradiated F_2, XeF_2, MF_2 (M = Cu, Ni) and metal A (A = Ru, Rh, Re, Os, Ir, Pt), AF_3 (A = Cr, Au), and AF_5 (M = Nb, Ta, As), respectively, in anhydrous HF. The products observed upon crystallization and the experimental conditions for the reactions between XeF_5SbF_6 and $M(SbF_6)_2$ (M = Sn, Pb) are also given.

Molar ratio of starting compounds A	: B	: C	Quantities used n(A) : n(B) : n(C) / mmol	V aHF / ml	p(F2) / bar	Dissolved in aHF[a]	Time of crystallization / days	Observed single crystals upon the crystallization[b]	Phases detected by Raman spectroscopy[c]
XeF_2	: CuF_2	: CrF_3	0.17 : 017 : 0.51	7	3	no	44	$(XeF_5CrF_5)_4{\cdot}XeF_4$	$(XeF_5CrF_5)_4{\cdot}XeF_4$
XeF_2	: CuF_2	: NbF_5	0.16 : 0.16 : 0.48	6	3	no	15	XeF_5NbF_6, $[XeF_5][Nb_2F_{11}]$	XeF_5NbF_6, $[XeF_5][Nb_2F_{11}]$
XeF_2	: CuF_2	: TaF_5	0.17 : 017 : 0.51	10	6	no	21	XeF_5TaF_6, TaF_5, $[XeF_5][Ta_2F_{11}]$	/
XeF_2	: NiF_2	: TaF_5	0.17 : 017 : 0.51	10	6	no	29	XeF_5TaF_6,TaF_5, H_3OTaF_6, $[XeF_5][Ta_2F_{11}]$	XeF_5TaF_6, $[XeF_5][Ta_2F_{11}]$
XeF_2	: CuF_2	: Ru	0.16 : 0.16 : 0.48	7	3	no	19	XeF_5RuF_6, O_2RuF_6	/
XeF_2	: NiF_2	: Ru	0.16 : 0.16 : 0.48	6	3	no	19	XeF_5RuF_6, O_2RuF_6	XeF_5RuF_6, O_2RuF_6
XeF_2	: CuF_2	: Rh	0.16 : 0.16 : 0.48	6	6	no	34	XeF_5RhF_6	XeF_5RhF_6
XeF_2	: NiF_2	: Rh	0.16 : 0.16 : 0.48	6	6	no		too small crystals	XeF_5RhF_6
XeF_2	: NiF_2	: Re	0.16 : 0.16 : 0.48	6	3	no	22	$(Xe_2F_{11})_2(NiF_6)$	/
XeF_2	: CuF_2	: Os	0.16 : 0.16 : 0.48	6	3	no	13	XeF_4	XeF_4, unknown phase
XeF_2	: NiF_2	: Os	0.16 : 0.16 : 0.48	6	3	no	22	too small crystals	/
XeF_2	: CuF_2	: Ir	0.17 : 0.17 : 0.51	7	3	no	32	XeF_5IrF_6	XeF_5IrF_6
XeF_2	: NiF_2	: Ir	0.16 : 0.16 : 0.48	6	3	no	10	XeF_5IrF_6, $Ni(XeF_2)_2(IrF_6)_2$	XeF_5IrF_6
XeF_2	: CuF_2	: Pt	0.16 : 0.16 : 0.48	6	6	no	34	XeF_5PtF_6	XeF_5PtF_6, O_2PtF_6
XeF_2	: NiF_2	: Pt	0.16 : 0.16 : 0.48	6	6	no	22	XeF_5PtF_6, O_2PtF_6	O_2PtF_6
XeF_2	: CuF_2	: AuF_3	0.2 : 0.2 : 0.6	3	3.5	no	24	XeF_5AuF_6	/
XeF_2	: NiF_2	: AuF_3	0.2 : 0.2 : 0.6	3	3.5	no	24	XeF_5AuF_6	/
XeF_2	: CuF_2	: AsF_5	0.35 : 0.35 : 1.05	5	4	yes	32	$CuFAsF_6$	XeF_5AsF_6
XeF_2	: NiF_2	: AsF_5	0.35 : 0.35 : 1.05	5	4	yes	32	$XeF_5Ni(AsF_6)_3$	$XeF_5Ni(AsF_6)_3$
XeF_5SbF_6	: $Sn(SbF_6)_2$		0.19 : 0.19	6	/	no	38	$XeF_5Sb_2F_{11}$	$XeF_5Sb_2F_{11}$, unknown phase
XeF_5SbF_6	: $Pb(SbF_6)_2$		0.17 : 0.17	4	/	yes	25	XeF_5SbF_6	XeF_5SbF_6 , unknown phase

[a]For crystallization, the clear supernatant containing no visible sediment was decanted into the side arm of the double-armed crystallization vessel.

[b]Phases that could be determined by single crystal X-ray diffraction. Too small single crystals and powdery material could not be identified by this method, of course.

[c]Phases that were detected by Raman spectroscopy.

Table S2. Experimental conditions and observed products upon crystallization in the experiments to prepare $XeF_5M(BF_4)_x(SbF_6)_{3-x}$ (x = 1, 2, 3; M = Co, Mn, Ni, Zn) salts.

Molar ratio of starting compounds A : B	Quantities used n(A) : n(B) / mmol	V aHF / ml	$p(BF_3)$ / bar	$p(F_2)$ / bar	Dissolved in aHF[a]	Time of crystallization / days	Desired product	Observed single crystals upon the crystallization[b]	Phases detected by Raman spectroscopy[c]
XeF_2 : $Zn(SbF_6)_2$	0.09 : 0.09	5	0.13	4.5	no	42	$XeF_5Zn(BF_4)(SbF_6)_2$	XeF_5SbF_6	/
XeF_5SbF_6 : CoF_2	0.35 : 0.35	8	1.2	/	yes	41	$XeF_5Co(BF_4)_2(SbF_6)$	XeF_5SbF_6	/
XeF_5SbF_6 : MnF_2	0.29 : 0.29	6	4	/	no	68	$XeF_5Mn(BF_4)_2(SbF_6)$	/	$[XeF_5][Sb_2F_{11}]$, $[XeF_5][SbF_6]$, MnF_3
XeF_5SbF_6 : NiF_2	0.31 : 0.31	5	0.6	4.5	no	42	$XeF_5Ni(BF_4)_2(SbF_6)$	XeF_5SbF_6	/
XeF_2 : NiF_2	0.40 : 0.40	6	1.3	4.5	no	46	$XeF_5Ni(BF_4)_3$	XeF_5BF_4	/

[a]For crystallization, the clear supernatant containing no visible sediment was decanted into the side arm of the double-armed crystallization vessel.

[b]Phases that could be determined by single crystal X-ray diffraction. Too small single crystals and powdery material could not be identified by this method, of course.

[c]Phases that were detected by Raman spectroscopy.

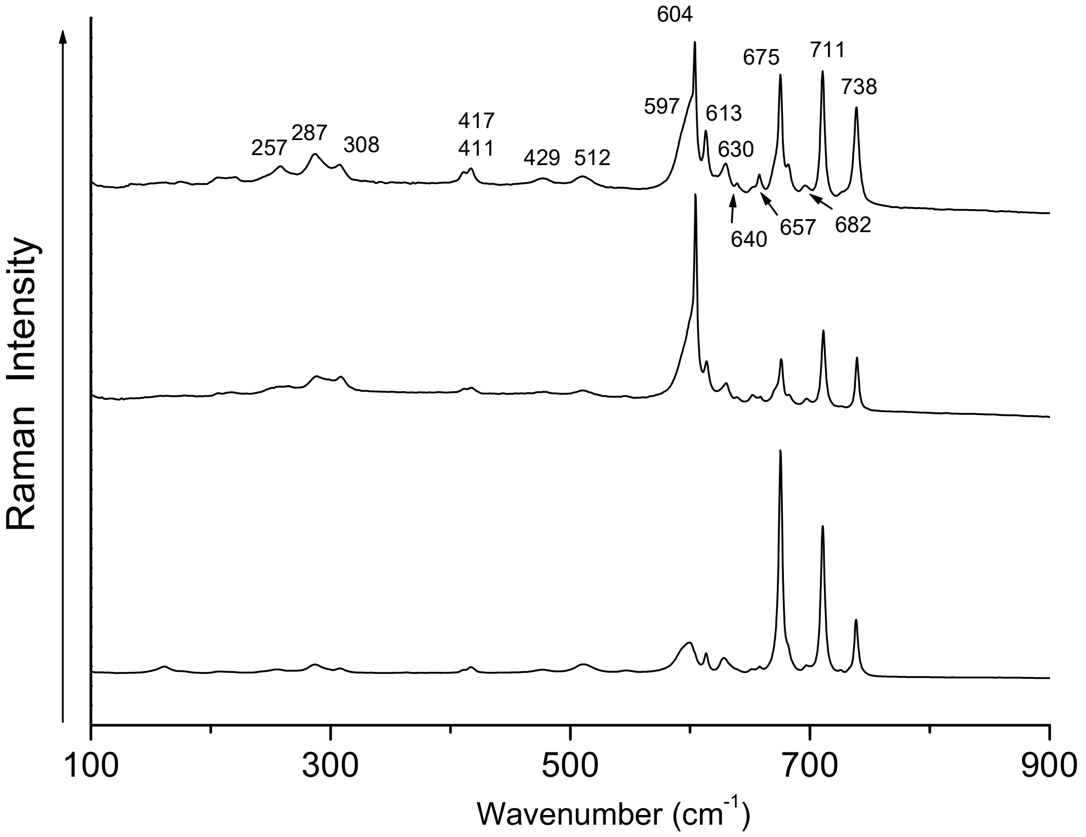

Figure S1. Raman spectra of XeF_5NbF_6 recorded on a single crystal.

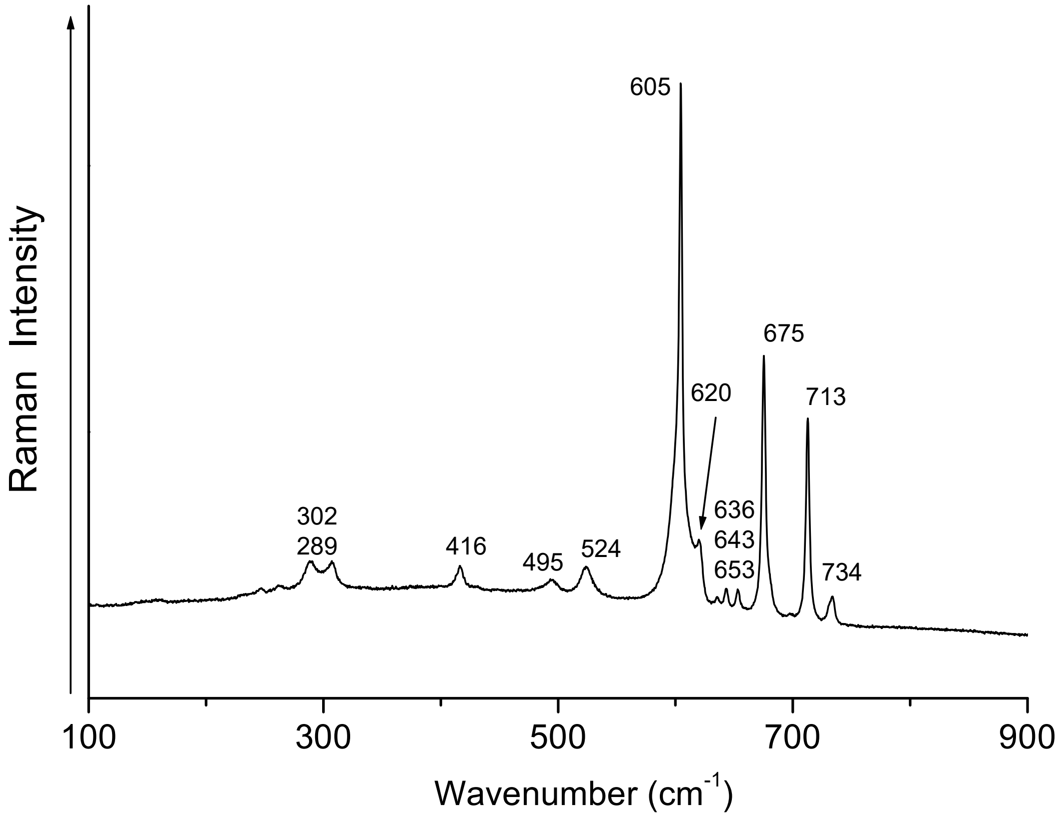

Figure S2. Raman spectra of XeF_5TaF_6 recorded on a single crystal.

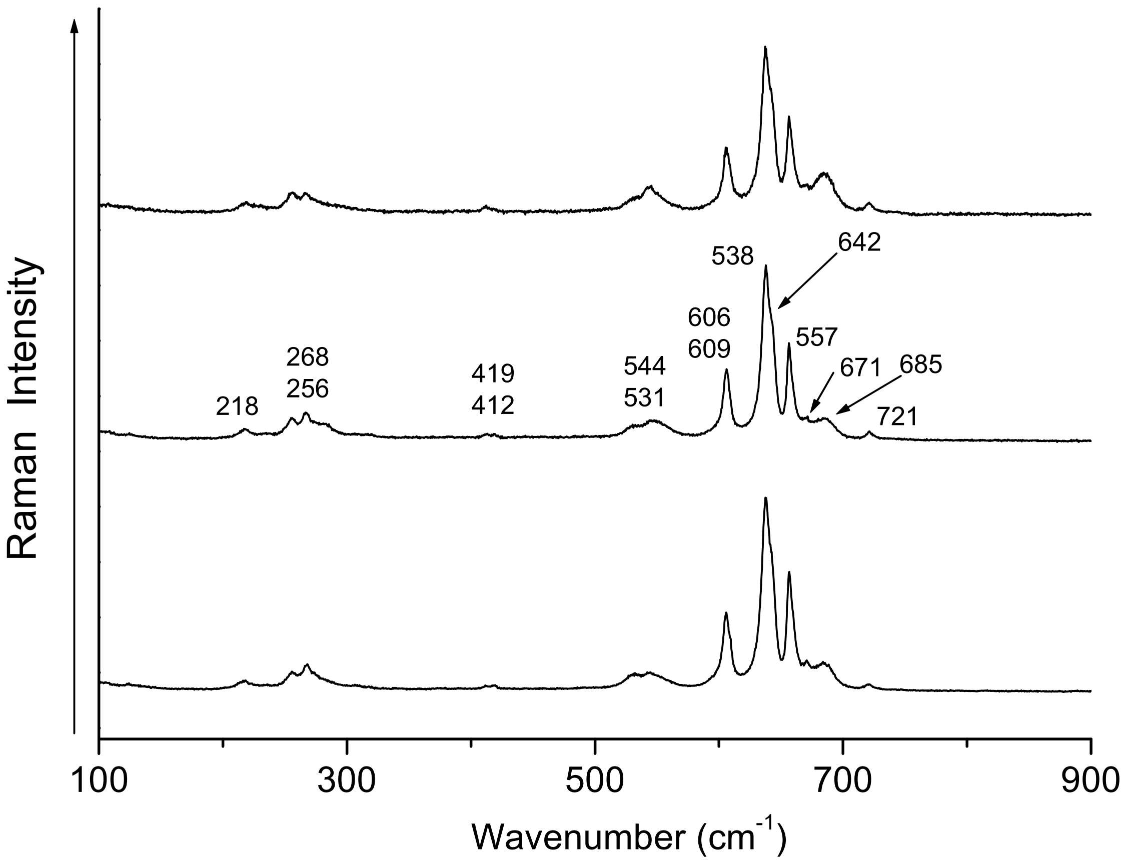

Figure S3. Raman spectra of XeF_5RhF_6 recorded on a single crystal.

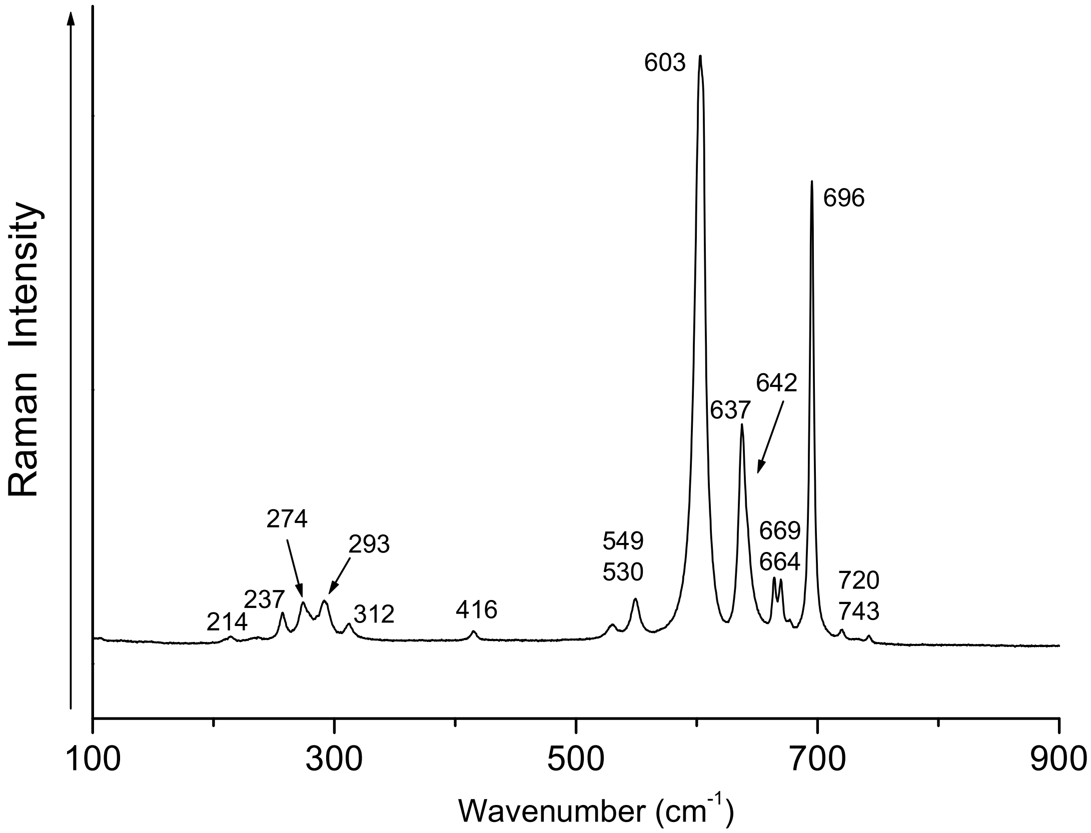

Figure S4. Raman spectra of XeF_5RuF_6 recorded on a single crystal.

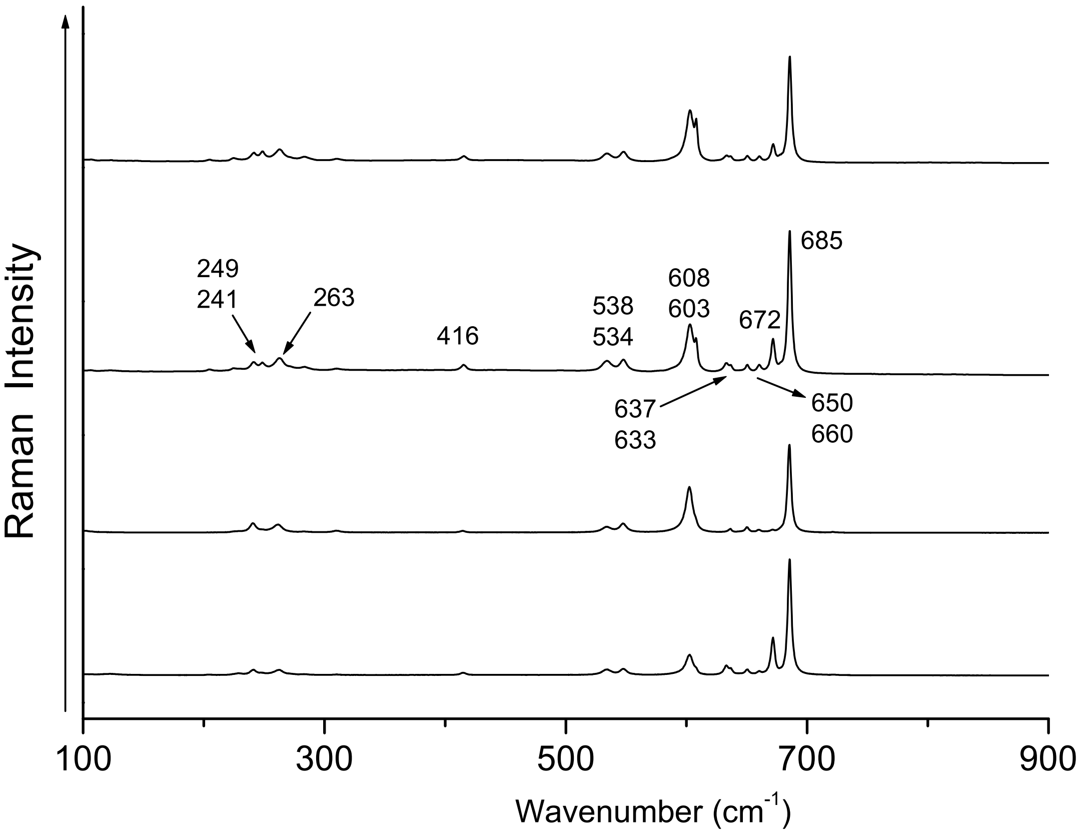

Figure S5. Raman spectra of XeF_5IrF_6 recorded on a single crystal.

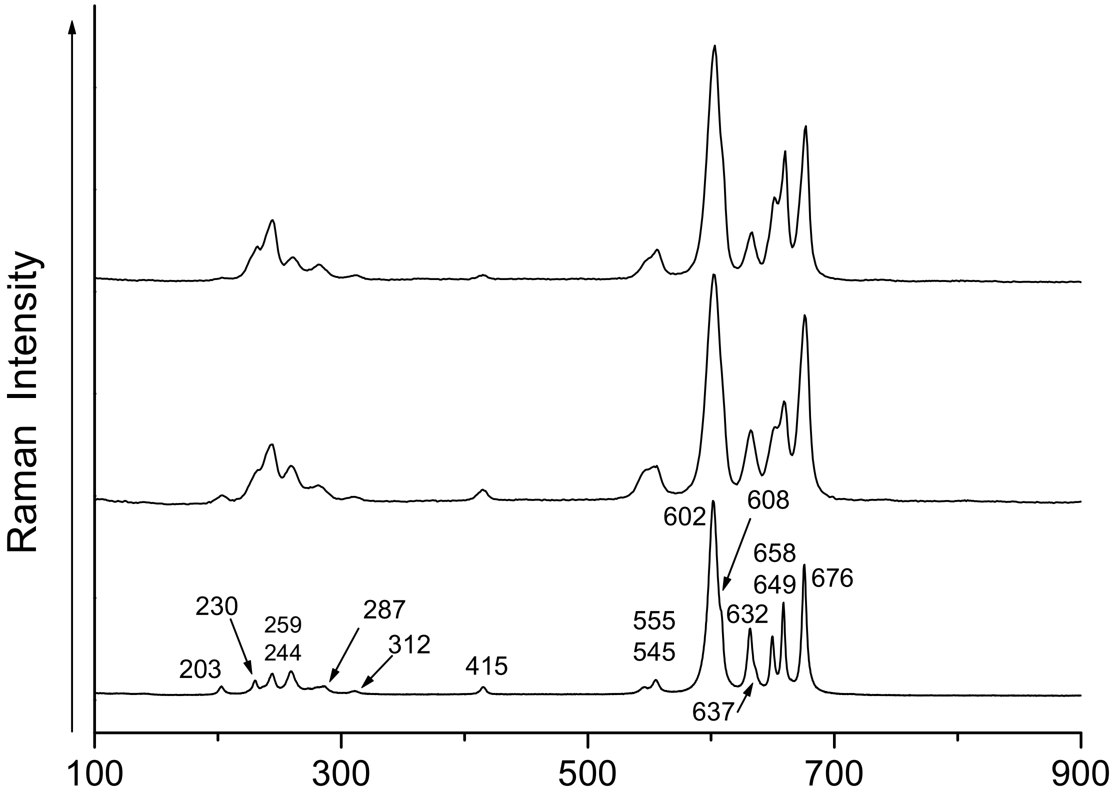

Figure S6. Raman spectra of XeF_5PtF_6 recorded on a single crystal.

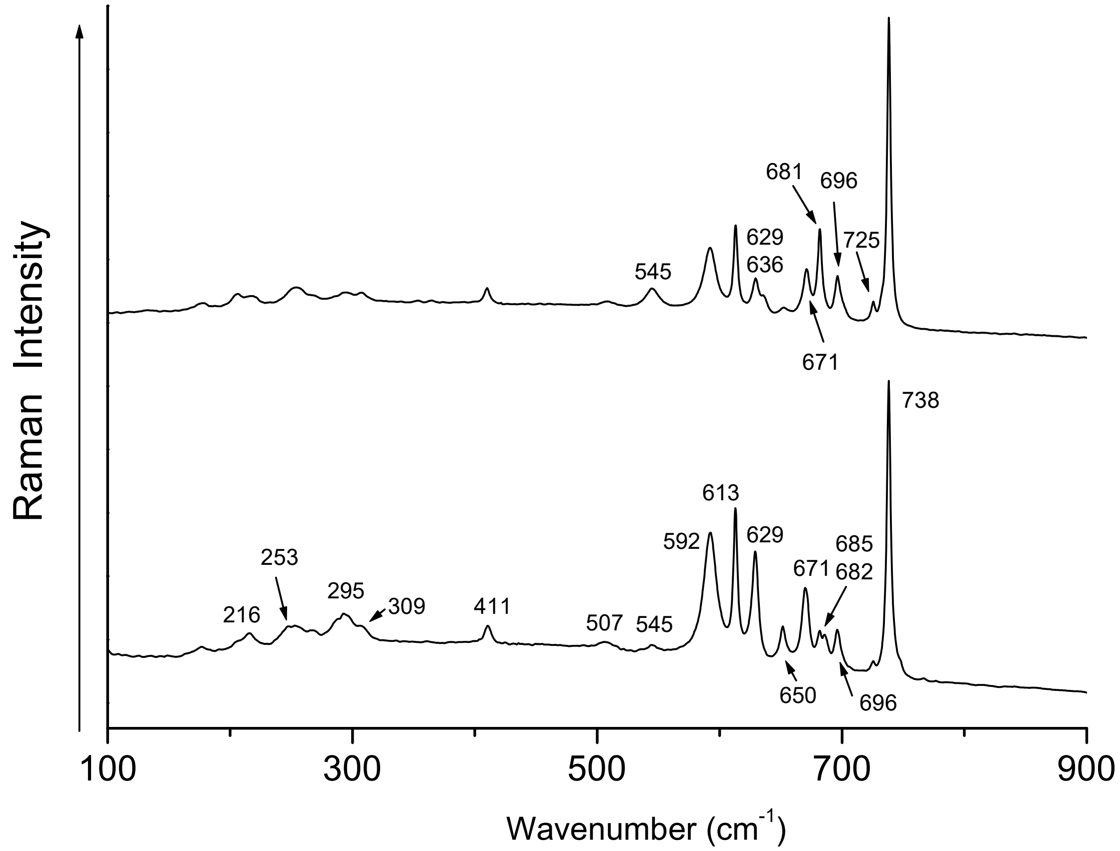

Figure S7. Raman spectra of $XeF_5Nb_2F_{11}$ recorded on a single crystal.

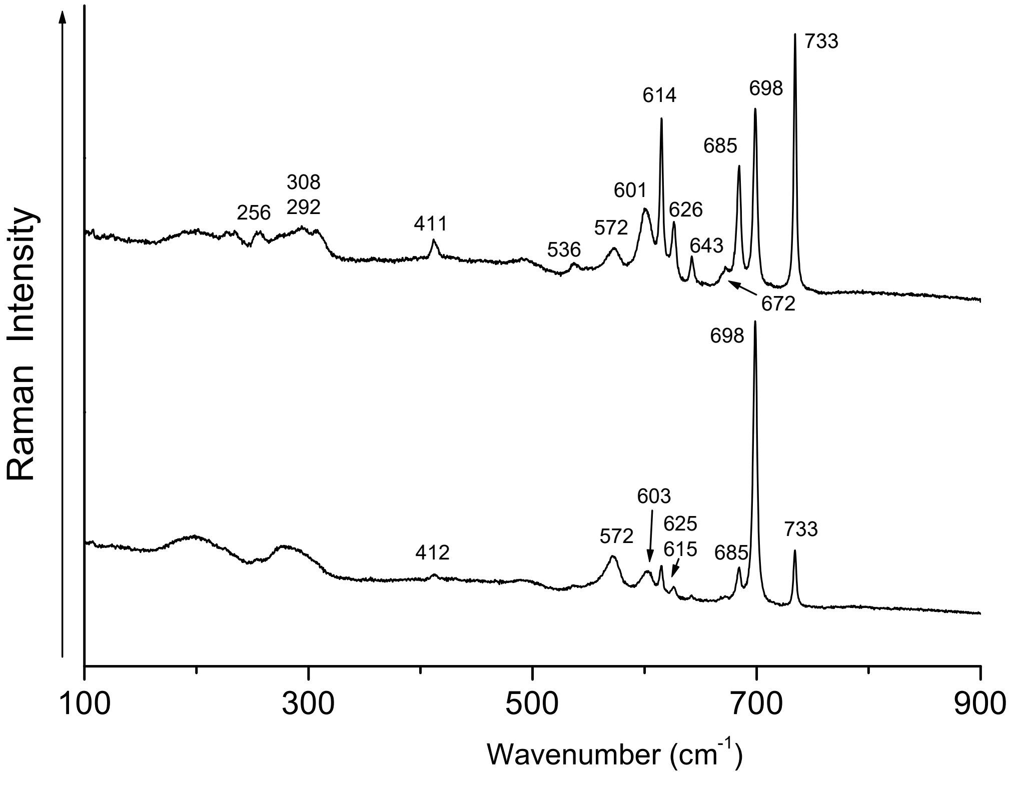

Figure S8. Raman spectra of $XeF_5Ta_2F_{11}$ recorded on a single crystal.

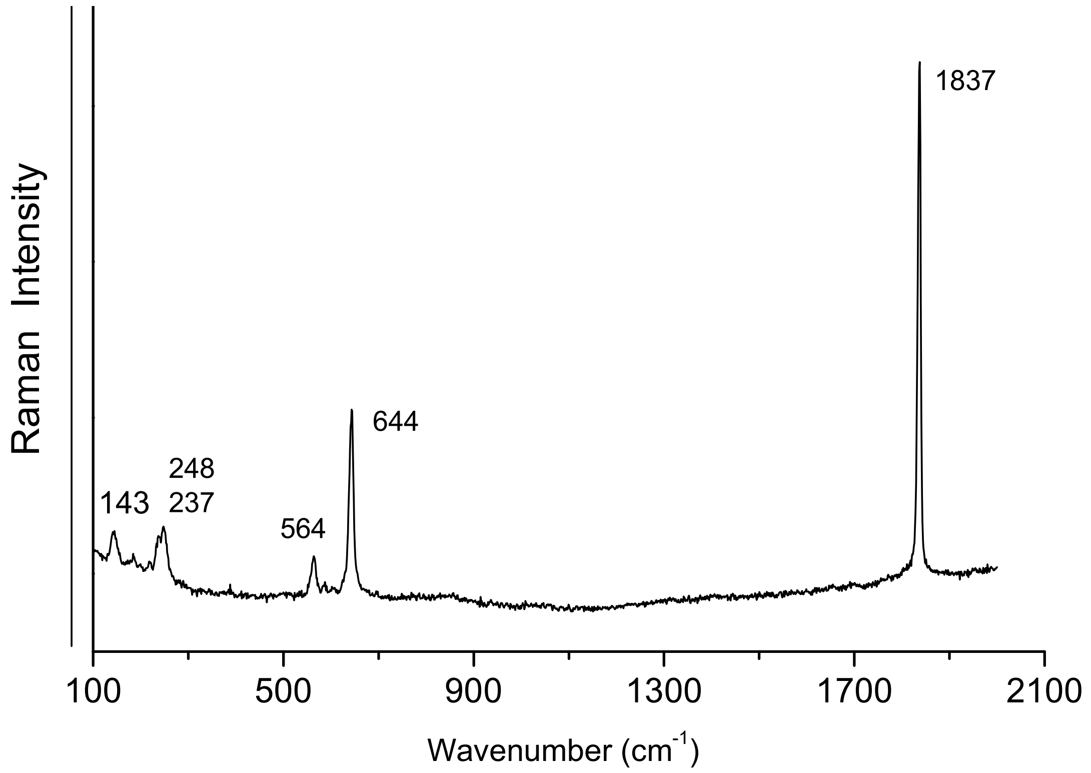

Figure S9. Raman spectra of O_2PtF_6 recorded on a single crystal.

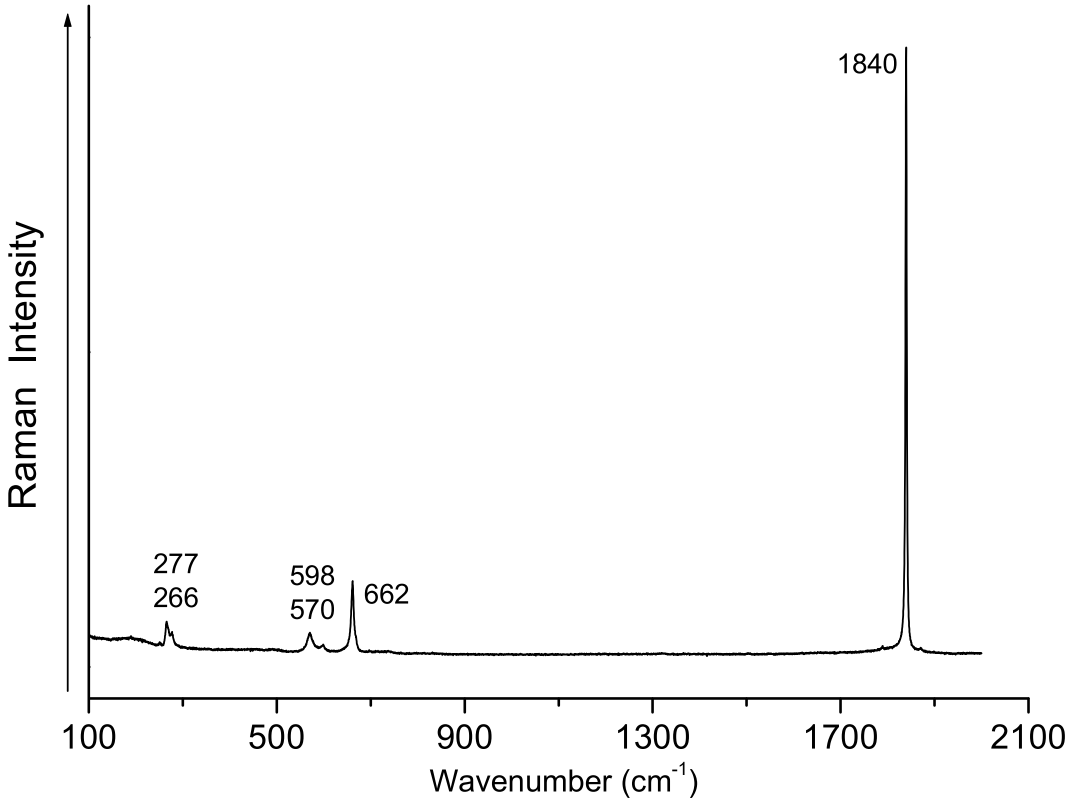

Figure S10. Raman spectra of O_2RuF_6 recorded on a single crystal.

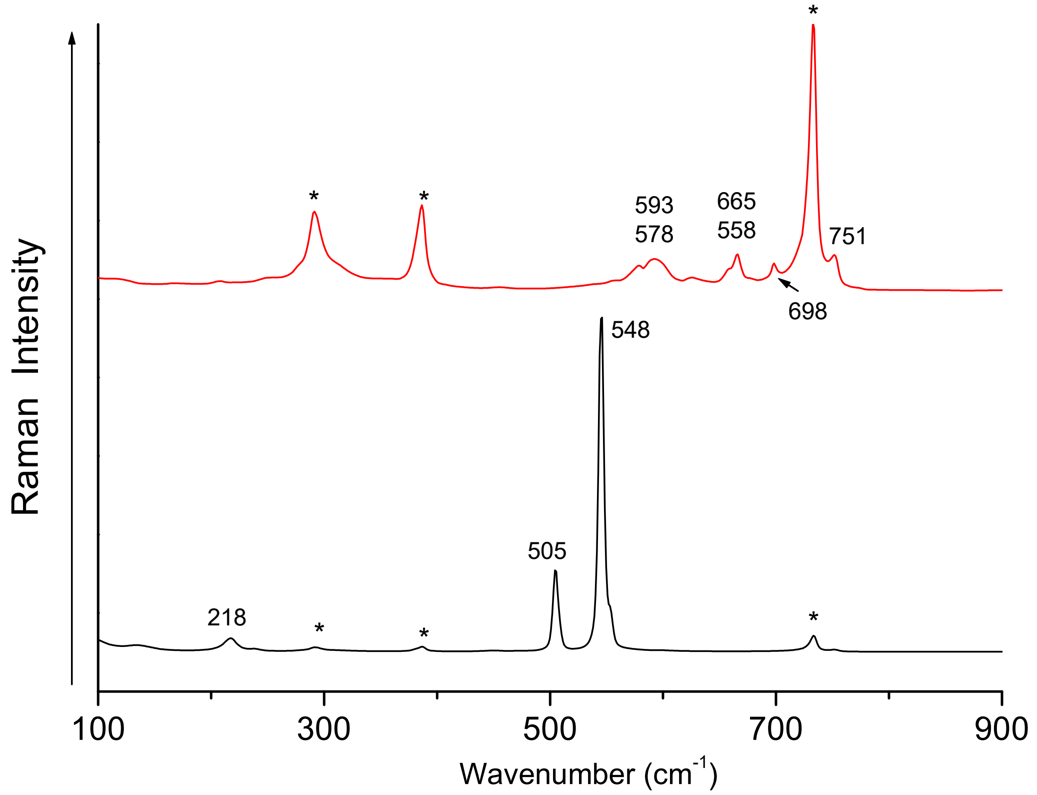

Figure S11. Raman spectra of single crystals after crystallization of the reaction product between XeF_2, Os powder and UV-irradiated F_2 in anhydrous HF: XeF4 (bottom), unknown product (top). An asterisk indicates vibrational bands of the perfluorinated polymer (FEP) of the reaction vessel.
